# The Feasibility of Home-Based Moxibustion for Fatigue in Peritoneal Dialysis: A Randomized Controlled Pilot Trial

**DOI:** 10.3390/healthcare14183110

**Published:** 2026-09-20

**Authors:** Yiqi Chen, Xiaoxuan Hu, Xiaoling Liang, Jianfeng Wu, Xiaoning Xie, Xiankun Chen, Fuhua Lu, La Zhang

**Affiliations:** 1 The Second Clinical Medical College of Guangzhou University of Chinese Medicine, Guangzhou 510405, China; 20231120939@stu.gzucm.edu.cn (Y.C.);; 2 Department of Nephrology, Guangdong Provincial Hospital of Chinese Medicine (The Second Affiliated Hospital of Guangzhou University of Chinese Medicine), Guangzhou 510120, China; 3 National Clinical Research Base of Traditional Chinese Medicine for Chronic Kidney Disease, Guangzhou 510120, China; 4 Key Unit of Methodology in Clinical Research, Guangdong Provincial Hospital of Chinese Medicine, Guangzhou 510120, China; 5 State Key Laboratory of Traditional Chinese Medicine Syndrome, Guangzhou 510006, China

**Keywords:** peritoneal dialysis, fatigue, moxibustion, randomized pilot trial, feasibility

## Abstract

**Objective**: Fatigue is a highly prevalent and debilitating symptom among patients undergoing peritoneal dialysis (PD). Yet, feasible home-based interventions remain limited. To assess the feasibility and observed safety of home-based moxibustion in patients receiving PD and to explore the preliminary effects on fatigue. **Methods**: This single-center, waitlist-controlled randomized pilot trial was conducted at a tertiary hospital in Southern China from February to July 2023. Adults receiving maintenance PD with persistent fatigue and an overall Revised Piper Fatigue Scale (PFS-R) score of 4 or higher were randomly assigned at a 1:1 ratio to either a moxibustion or a waitlist control group. Participants in the intervention group received home-based moxibustion at the Shenque (CV8) acupoint at least 3 times per week for 12 weeks, in addition to standard PD care. Control participants received standard care alone during the trial and were offered delayed moxibustion supplies after completion of the last follow-up. The prespecified primary outcomes were the between-group difference in the mean overall PFS-R score at week 12 and the proportion of participants in the moxibustion group who achieved the target intervention dose. Additional outcomes included study retention, completion of scheduled study contacts, secondary clinical outcomes, and adverse events. **Results**: Of the 262 patients assessed for eligibility, 44 met the eligibility criteria, of whom 25 declined participation for personal reasons, and 19 were randomized (mean [SD] age, 47.5 [12.4] years; 16 [84.2%] women). All completed the 12-week follow-up. All 57 scheduled on-site visits and 56 of 57 telephone follow-ups (98.2%) were completed. Eight of nine participants in the moxibustion group achieved the target intervention dose (88.9%; 95% CI, 51.8–99.7%). The exploratory fatigue findings were inconsistent. In the exploratory week-12 comparison, the mean PFS-R score was lower in the moxibustion group than in the control group (unadjusted mean difference, −1.09 points; 95% CI, −2.15 to −0.03; *p* = 0.04). However, the between-group difference in change from baseline was −0.36 points (95% CI, −2.03 to 1.31; *p* = 0.65), and no group-by-time interaction was observed (*p* = 0.86). Other clinical outcomes showed no consistent between-group differences. Adverse events occurred in four of nine participants in the moxibustion group and two of ten in the control group. No serious adverse events or permanent intervention discontinuations occurred. **Conclusions and Relevance**: Among selected randomized participants, a 12-week home-based moxibustion intervention supported by standardized training and follow-up was delivered with high adherence and complete retention. However, the low screening-to-randomization proportion limits conclusions about recruitment feasibility and broader implementation, and the exploratory findings do not establish efficacy or provide definitive evidence of safety. Further evaluation in a larger, sham-controlled trial is warranted.

## 1. Introduction

Peritoneal dialysis (PD) is a widely used renal replacement therapy for patients with end-stage kidney disease (ESKD), accounting for approximately 11% of the global dialysis population [1]. Compared with hemodialysis, PD may offer more treatment flexibility, lower healthcare resource utilization, and better preservation of residual kidney function [2]. Nevertheless, patients receiving PD experience a high symptom burden [3], and fatigue is among the most common and debilitating symptoms, affecting over 70% of this population [4,5]. Fatigue in patients receiving PD is multifactorial and may be associated with uremic toxin accumulation, inflammation, anemia, malnutrition, and/or psychological distress [6,7]. It is also independently associated with poorer quality of life, an increased risk of hospitalization, and higher mortality [8].

Given its substantial contribution to impaired quality of life among patients receiving dialysis, fatigue represents an important target for supportive care. Although exercise and cognitive behavioral therapy have shown promise, their implementation may require multidisciplinary support, trained personnel, and additional healthcare resources. These requirements may limit their accessibility and sustained use, particularly among patients receiving PD at home and in resource-limited settings [9]. Therefore, practical, resource-efficient interventions that can be integrated into home-based care warrant investigation.

Moxibustion is a traditional therapy in which dried mugwort (*Artemisia*) is burned near specific acupoints to provide stimulation [10]. Its relatively simple procedure and potential for self-administration after appropriate training may make it suitable for home-based supportive care. Experimental studies have proposed several mechanisms, including modulation of the autonomic nervous system [11], improvement of microcirculation [12], and attenuation of systemic inflammation [13]. Yet, clinical evidence for its effects on fatigue remains preliminary, with studies conducted in patients with cancer [14] or CKD [15] and patients receiving hemodialysis [16].

However, to our knowledge, no previous studies have evaluated home-based moxibustion for fatigue in patients receiving PD. Thus, uncertainties remain regarding whether patients with fatigue can be recruited and retained, can be trained to perform moxibustion at home, and whether they are able to complete the prescribed treatment over 12 weeks. The completeness of outcome data and adverse events observed during home use also require characterization before a larger trial is undertaken. Accordingly, we conducted a pilot randomized controlled trial of home-based moxibustion for fatigue in patients receiving PD. This report focuses on recruitment, retention, treatment delivery, adherence, data completeness, and observed adverse events, while changes in fatigue and other clinical outcomes were also explored to inform the design of a future trial.

## 2. Methods

### 2.1. Study Design

This was a single-center, two-arm, waitlist-controlled pilot randomized controlled trial conducted at the peritoneal dialysis center at Guangdong Provincial Hospital of Chinese Medicine, a tertiary hospital in southern China. Participants completed a 1-week run-in phase, followed by a 12-week intervention period. They were enrolled between 17 February and 17 April 2023, and the final follow-up was completed on 19 July 2023.

The study protocol was approved by the Ethics Committee at Guangdong Provincial Hospital of Chinese Medicine (No. BF2022-229). The trial was registered with the Chinese Clinical Trial Registry (ChiCTR2300070682). We initially submitted the trial registration to the Chinese Clinical Trial Registry on 8 October 2022 and, in accordance with ethical requirements, filed the study in the Chinese Medical Research Registration and Record Filing Information System on 17 November 2022. Due to a registry system malfunction, the original submission was not captured in the public database; as instructed by the registry, we resubmitted the record, which now appears online with a later, retrospectively assigned date of 20 April 2023. For transparency, the first patient was enrolled on 20 February 2023—after the initial submission but before the currently displayed registry date. The IRB-approved prespecified protocol (version dated 29 September 2022, Supplement S1) predates enrollment. The trial was reported in accordance with the Consolidated Standards of Reporting Trials (CONSORT) reporting guidelines [17,18].

### 2.2. Patients

Participants were recruited from the peritoneal dialysis outpatient clinic and the nephrology inpatient wards. Eligible participants were adults aged 18 years or older who had maintained peritoneal dialysis for at least 4 months, were clinically stable, and had experienced fatigue for at least 4 weeks, with an overall Revised Piper Fatigue Scale (PFS-R) score of 4 or higher. Exclusion criteria included previous kidney transplantation or concurrent hemodialysis; fatigue attributable to acute or reversible medical conditions including severe anemia, active infection, acute exacerbation of heart failure, malnutrition, or other unstable comorbidities requiring recent adjustment of dialysis prescription; and contraindications to or intolerance of moxibustion. The full eligibility criteria are provided in Supplemental Table S1. All participants provided written informed consent.

### 2.3. Randomization and Masking

Participants were randomly assigned at a 1:1 ratio to either the moxibustion group or the waitlist control group using a computer-generated block randomization sequence implemented through a centralized, web-based allocation system. This system was developed and overseen by the hospital’s independent methodology department. The allocation sequence remained concealed from the enrolling investigators until the participant had completed the run-in phase, remained eligible and was ready for randomization. The randomization sequence and block size were generated by a statistician who was not involved in participant enrollment, data collection, or outcome assessment. Group assignments were released through the system to a designated study coordinator responsible for implementing allocation. Because of the nature of the intervention, the participants and personnel involved in intervention delivery were not masked to group assignment. Statistical analyses were performed by a statistician masked to the group labels.

### 2.4. Intervention Procedures

During the 1-week run-in phase, all participants received individual training on how to use the moxibustion device and complete the treatment diary. Procedural competency was confirmed before randomization. Participants assigned to the moxibustion group retained the device and supplies and received home-based moxibustion in addition to standard PD care. Moxibustion was applied to the Shenque (CV8) acupoint, located at the center of the umbilicus. Each session lasted at least 15 min and was performed at least 3 times per week for 12 weeks using a standardized moxibustion device (manufactured by Aijiangshan Health Technology Qichun Medical Co., Ltd., Guangzhou, China). Participants were instructed to adjust the heat intensity according to a comfortable sensation of local warmth or mild skin flushing. The structure and application of the moxibustion device are shown in Supplemental Figures S1–S3. Treatment adherence was assessed at each on-site visit by cross-checking treatment diary entries against the returned unused moxa cones.

Participants assigned to the waitlist control group returned the moxibustion devices and supplies after randomization and continued standard PD management alone during the 12-week study period. Standard care included routine clinical follow-up and clinically indicated adjustments to the dialysis prescription and concomitant medications. Participants were asked not to initiate interventions specifically intended to relieve fatigue, including acupuncture, medication, or physical therapy. After completing the week-12 visit, their moxibustion devices and supplies were returned to them to undertake the same home-based intervention. A waitlist design, rather than sham control, was used because no validated sham moxibustion device suitable for unsupervised home use was available.

Both groups followed the same schedule for study contacts. All participants were scheduled to receive three telephone follow-ups and three on-site visits during the 12-week intervention phase.

### 2.5. Outcomes

The prespecified primary outcomes were the between-group difference in mean overall Revised Piper Fatigue Scale (PFS-R) scores [19] at week 12 and the proportion of participants in the moxibustion group who achieved the target intervention dose. The PFS-R is a validated 22-item instrument with overall scores ranging from 0 to 10, categorized as no (0), mild (1–3), moderate (4–6), or severe fatigue (7–10). The target intervention dose was defined as completion of at least three moxibustion sessions in each week of the 12-week intervention period. Additional feasibility measures included retention, completion of scheduled follow-ups and visits, the number of completed sessions, and dose intensity relative to the minimum prescribed dose.

Secondary outcomes included laboratory parameters related to ESKD, dialysis adequacy, nutritional status, sleep quality (Pittsburgh Sleep Quality Index [PSQI]), physical activity (International Physical Activity Questionnaire—Short Form [IPAQ-SF]), anxiety and depression symptoms (Hospital Anxiety and Depression Scale [HADS]), and overall quality of life (European Organization for Research and Treatment of Cancer Quality of Life Questionnaire Core 30 [EORTC QLQ-C30]). Patient-reported outcomes were assessed at baseline and weeks 4, 8, and 12; laboratory, dialysis adequacy, and nutritional measures were assessed at baseline and week 12. Further details are provided in Supplemental Table S2.

Adverse events were monitored throughout the intervention period, including the number of affected participants and events, severity and seriousness, relationship to the intervention, and any resulting interruption or discontinuation. Questionnaires were completed during on-site visits, and laboratory parameters were extracted and recorded from electronic medical records by researchers not involved in delivering the intervention.

### 2.6. Statistical Analysis

This pilot trial was designed primarily to assess feasibility and obtain preliminary estimates of the treatment effect. Based on a previous study [20] in which the mean (SD) fatigue score was 3.77 ± 0.50 in the intervention group and 4.88 ± 0.61 in the control group, 8 participants per group were required when using PASS software version 11.0 (NCSS LLC, Kaysville, UT, USA), with a two-sided α of 0.05 and 90% power. After allowing for a 20% attrition rate, the target sample size was 20 participants (10 per group). This sample size was intended to support the pilot objectives rather than definitive efficacy testing.

All randomized participants were analyzed according to their assigned groups. Statistical analyses were conducted by an independent statistician blinded to group allocation using Stata version 11.0 (StataCorp, College Station, TX, USA) and R version 4.4.3 (R Foundation Statistical Computing, Vienna, Austria). Feasibility outcomes were summarized descriptively, with an exact binomial 95% CI calculated for target-dose achievement. The week-12 PFS-R scores were compared using an independent-samples *t* test, and changes over time were evaluated using repeated-measures ANOVA. Fatigue severity categories were compared using Fisher’s exact test, and between-group differences in change from baseline were examined post hoc. An exploratory post hoc analysis of covariance adjusted the week-12 PFS-R comparison for baseline PFS-R score.

Secondary continuous outcomes were summarized as mean (SD) and compared using independent-samples *t* tests when approximately normally distributed; otherwise, they were summarized as median (IQR) and compared using Mann–Whitney *U* tests. Between-group effects were reported as mean differences with 95% CIs for approximately normally distributed outcomes and as Hodges–Lehmann location-shift estimates with 95% CIs for nonnormally distributed outcomes. Categorical variables were compared using Fisher’s exact tests. Within-group changes and repeated patient-reported outcomes were analyzed using paired tests or repeated-measures analysis of variance. Adverse events were compared using Fisher’s exact tests, and event counts were summarized descriptively. Effect estimates are presented with 95% CIs. No adjustment was made for multiple comparisons because analyses were exploratory. All tests were two-sided with *p* < 0.05 considered statistically significant.

## 3. Results

### 3.1. Participants

**Figure 1:**
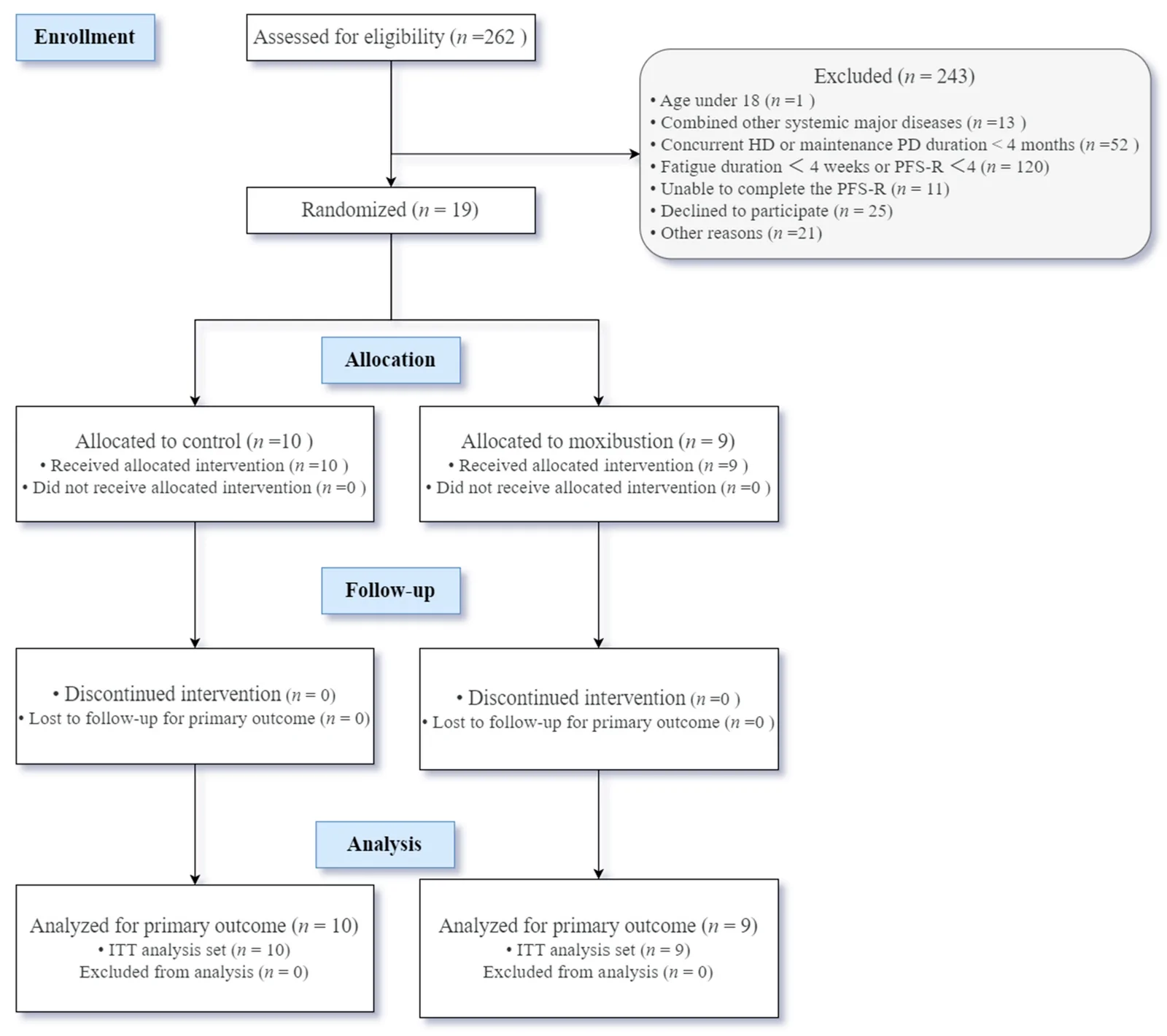
CONSORT flow diagram. Figure legend: Abbreviations: HD, hemodialysis; PFS-R, Revised Piper Fatigue Scale; ITT, intention-to-treat.

A total of 262 patients were assessed for eligibility. Of these, 218 did not meet the eligibility criteria, most commonly because of insufficient fatigue duration or severity, concurrent hemodialysis, or maintenance PD for less than 4 months. Among the 44 patients who met the eligibility criteria, 25 (56.8%) declined participation for personal reasons and 19 participants (43.2%) consented to participate and were randomized, corresponding to 7.3% of all patients screened. Ten were assigned to the waitlist control group and nine to the moxibustion group. All 19 participants completed the 12-week follow-up and were included in the intention-to-treat analysis (Figure 1).

Several between-group differences in baseline characteristics were observed (Table 1). Compared with the control group, participants in the moxibustion group were older and had higher body mass index (BMI), Charlson Comorbidity Index (CCI), and C-reactive protein levels, but lower anxiety scores. Participants in the control group had a longer PD duration and a higher baseline PFS-R score, whereas those in the moxibustion group had higher residual kidney Kt/V and residual kidney creatinine clearance.

### 3.2. Feasibility Outcomes

All 19 randomized participants completed the 12-week follow-up and provided primary outcome data at the end of the study, corresponding to a study retention rate of 100%. All 57 scheduled on-site visits were completed, and 56 of 57 scheduled telephone follow-ups were completed (98.2%). All nine participants assigned to the moxibustion group completed the intervention period. Eight participants achieved the target moxibustion dose (88.9%; 95% CI, 51.8% to 99.7%). The mean number of moxibustion sessions per participant was 50.4 (95% CI, 36.7 to 64.2). Overall, 454 sessions were completed, corresponding to 140.1% of the aggregate minimum target of 324 sessions.

### 3.3. Fatigue Outcome

**Figure 2:**
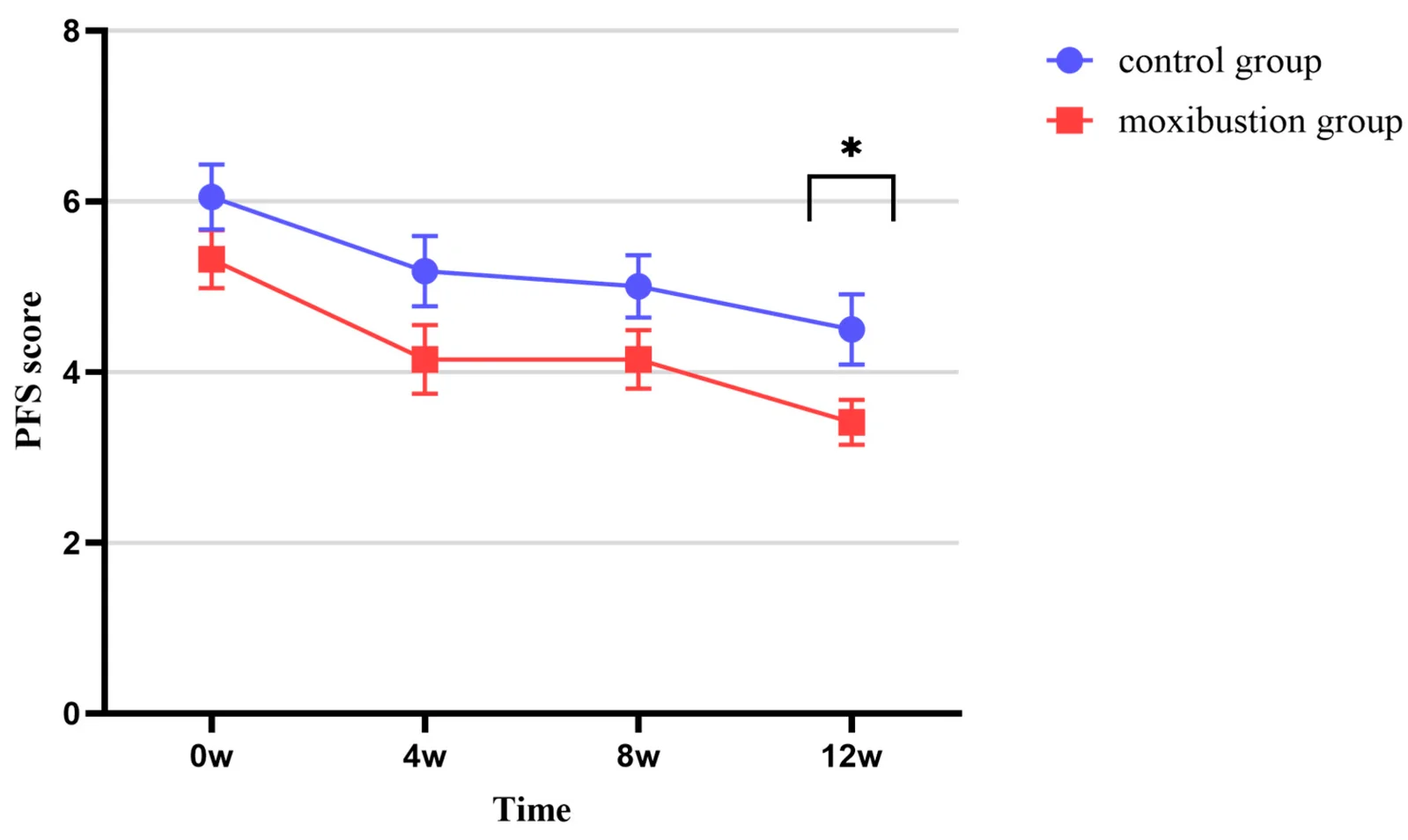
Changes in PFS-R outcomes over time by group. Figure legend: Data are presented as means, with error bars indicating standard errors. The asterisk indicates *p* < 0.05 for the between-group comparison at week 12 using an independent-samples *t* test. Abbreviation: PFS-R, Piper Fatigue Scale-Revised Scores.

In the exploratory week-12 comparison, the mean PFS-R score was lower in the moxibustion group than in the control group, with an unadjusted mean difference (MD) of −1.09 points (95% CI, −2.15 to −0.03; *p* = 0.04). A post hoc analysis adjusting for baseline PFS-R score yielded an adjusted between-group MD of −1.24 points (95% CI, −2.37 to −0.11; *p* = 0.03). However, the between-group difference in change from baseline was −0.36 points (95% CI, −2.03 to 1.31; *p* = 0.65), and no group-by-time interaction was observed in the repeated-measures ANOVA (*F* = 0.16; *p* = 0.86; Figure 2, Table 2, Supplemental Tables S4 and S8). Fatigue severity distributions at week 12 did not differ between groups (*p* = 0.65).

### 3.4. Secondary Outcomes

At week 12, the total creatinine clearance (CCr) was higher in the moxibustion group than in the control group (68.43 ± 19.89 vs. 47.64 ± 8.06 L/week; *p* = 0.007), with an unadjusted mean difference of 20.79 L/week (95% CI, 6.39 to 35.19; *p* = 0.007) (Table 2). However, the between-group difference in change from baseline was 6.26 L/week (95% CI, −10.38 to 22.89; *p* = 0.44). Changes in residual kidney CCr and peritoneal CCr also showed no clear between-group differences (Supplemental Tables S6 and S7). Additionally, no clear between-group differences in change were observed for the other dialysis adequacy measures.

No group-by-time interactions were observed for quality of life, sleep quality, physical activity, anxiety, or depression (all interaction *p*-values ≥ 0.23; Supplemental Figure S3, Tables S5 and S8). The remaining laboratory and nutritional outcomes are reported in Supplemental Tables S3, S6 and S7. All secondary outcome analyses were exploratory.

### 3.5. Adverse Events

During the 12-week intervention period, seven adverse events were recorded among four of nine participants (44.4%) in the moxibustion group, and four events were recorded among two of ten participants (20.0%) in the control group (Table 3). One case of constipation was considered related to moxibustion; treatment was temporarily interrupted and resumed once the event resolved. No serious adverse events or permanent intervention discontinuations due to adverse events occurred.

## 4. Discussion

Among the selected participants who were randomized, the 12-week home-based moxibustion was delivered with complete retention, high follow-up completion, and high target-dose achievement. Although the moxibustion group had a lower PFS-R score at week 12 in the unadjusted comparison, neither the between-group difference in change from baseline nor the group-by-time interaction showed clear evidence of benefit. Other clinical outcomes also showed no consistent between-group differences, and no serious adverse events or permanent intervention discontinuations occurred. These findings support further evaluation in a larger, sham-controlled trial but do not establish efficacy or definitive safety.

Nonpharmacological interventions, particularly exercise-based approaches, may mitigate fatigue in dialysis patients, although most evidence is derived from hemodialysis populations and remains of low certainty [9,21]. Evidence specific to PD is limited, and participation in physical activity may be constrained by reduced cardiopulmonary reserve, fluctuating symptoms, and/or kinesiophobia [22,23,24,25]. A recent patient-led workshop highlighted the need for accessible, individualized, home-based interventions that can accommodate varying energy levels and comorbidities [26]. These findings indicate that, among those who enrolled, the self-administered moxibustion was successfully delivered alongside standard PD care, with high adherence and retention. Thus, moxibustion should be considered a candidate adjunct for further evaluation rather than an established alternative to exercise-based rehabilitation.

Several studies have suggested that moxibustion may improve fatigue symptoms in patients with cancer-related fatigue and chronic fatigue syndrome [27,28]. Proposed mechanisms have included increasing metabolic heat production, modulating the hypothalamic–pituitary–adrenal (HPA) axis, regulating hippocampal neuropeptide expression, and improving autonomic nervous system function, although these mechanisms remain incompletely understood [29,30,31,32]. In the present trial, the lower week-12 PFS-R score in the unadjusted comparison was not supported by any clear between-group differences in change from either baseline or longitudinal trajectories. Therefore, these findings do not establish any specific anti-fatigue effect or mechanism. Although total creatinine clearance was higher in the moxibustion group at week 12, there was no clear between-group difference in change from baseline, and residual kidney clearance was already higher in this group at baseline. Thus, the isolated end-of-treatment difference may reflect baseline imbalance or sampling variability and should not be interpreted as a treatment effect.

This pilot randomized trial has several strengths. Its prospective, randomized design specifically evaluated the feasibility of self-administered, home-based moxibustion among patients undergoing PD. Intervention delivery was standardized through the use of a uniform device, individualized training, and competency assessment, while adherence was evaluated by cross-checking participant diaries against remaining moxa cone counts. Complete follow-up and systematic adverse-event monitoring provided information on retention, adherence, and initial safety. In addition, repeated patient-reported assessments together with laboratory and dialysis-related measures provided preliminary estimates that may inform outcome selection and study planning for a future adequately powered trial.

This study has several limitations. Firstly, the small sample size limited statistical precision and led to chance imbalances in several baseline characteristics; the moxibustion group was older and had higher CCI levels, but also had a shorter PD duration, higher residual kidney clearance, and numerically lower baseline anxiety and PFS-R scores. These differences could have influenced fatigue outcomes in opposing directions; although the exploratory analysis adjusted for baseline PFS-R score, the sample was too small for reliable multivariable adjustment, and their net impact could not be determined. The predominance of women (16 of 19 participants) further limits generalizability and precludes sex-based analyses. The study was not powered to confirm efficacy or detect uncommon adverse events, and the multiple clinical outcome analyses were exploratory without adjustment for multiplicity. The low screening-to-randomization proportion and the substantial proportion of eligible patients who declined participation limit conclusions regarding recruitment feasibility and broader implementation. Secondly, the waiting list control did not control for nonspecific attention, expectancy, placebo, or heat effects, and participants could not be blinded. Because fatigue and several outcomes were self-reported, knowledge of treatment assignment may have introduced reporting bias. Although sham moxibustion approaches, including infrared devices, have been developed, achieving credible blinding remains challenging, particularly among individuals familiar with moxibustion [33,34,35]. Thirdly, treatment intensity was guided by subjective thermal tolerance without precise temperature control, smoke-exposure monitoring, or filtration of combustion byproducts, limiting dose standardization and the assessment of longer-term safety [36,37]. Finally, although the PFS-R has been used in dialysis populations, it might not fully capture fatigue experiences specific to PD [38,39,40]. Some potentially relevant contributors to fatigue, including thyroid dysfunction, were not measured, and therefore could not be accounted for in the exploratory analyses.

These findings provide practical feasibility estimates for planning a future adequately powered trial. Such a study should prespecify stratification for key baseline factors such as fatigue severity, comorbidity burden and residual kidney function; incorporate a credible sham or attention-matched control; blind the outcome assessors and data analysts where feasible; objectively monitor treatment exposure and relevant device parameters; and include longer follow-up to assess the durability of clinical outcomes. Clinical efficacy and longer-term safety should be established before routine clinical implementation.

## 5. Conclusions

Among selected randomized patients undergoing PD, a 12-week home-based moxibustion intervention supported by standardized training and follow-up was delivered with complete retention, high completion of scheduled contacts, and high target-dose achievement. However, the low screening-to-randomization proportion limits conclusions regarding recruitment feasibility and broader implementation. Its effects on fatigue and other clinical outcomes, as well as its longer-term safety, remain uncertain and require evaluation in a larger, adequately powered, sham-controlled trial.

## Figures and Tables

**Table 1:** Baseline characteristics.

	Control Group (*n* = 10)	Moxibustion Group (*n* = 9)	*p* Value ^ab^
**Baseline information**			
Age, mean (SD), y	42.70 (8.58)	54.56 (10.41)	0.02
Gender, No. (%)			
Male	1 (10)	2 (22)	0.58
Female	9 (90)	7 (78)	
MAP ^c^, mean (SD), mmHg	99.73 (8.40)	108.41 (16.75)	0.17
CCI, median (IQR)	2.00 (2.00, 4.00)	5.00 (3.00, 6.00)	0.02
Time on dialysis, median (IQR), months	25.50 (16.00, 49.00)	19.00 (13.00, 26.00)	0.29
BMI, mean (SD), kg/m^2^	20.43 (2.37)	23.46 (2.10)	0.01
**Dialysis-Related Index**			
4-h D/P, mean (SD)	0.61 (0.10)	0.63 (0.14)	0.77
Total Kt/V ^d^, median (IQR)	2.14 (1.73, 2.29)	1.98 (1.76, 2.27)	0.90
Residual kidney Kt/V, median (IQR)	0.22 (0.00, 0.68)	0.38 (0.18, 0.74)	0.22
Peritoneal Kt/V, mean (SD)	1.76 (0.46)	1.53 (0.29)	0.22
Total CCr ^e^, median (IQR), L/week	51.02 (45.24, 56.05)	52.83 (51.95, 60.40)	0.10
Residual kidney CCr, mean (SD), L/week	11.63 (2.12)	25.00 (23.97)	0.14
Peritoneal CCr, mean (SD), L/week	38.12 (7.40)	39.27 (8.44)	0.76
**Laboratory Parameters**			
Serum creatinine, mean (SD), mg/dL	10.64 (2.09)	9.67 (3.29)	0.45
Serum urea nitrogen, mean (SD), mg/dL	57.10 (12.19)	64.52 (17.74)	0.30
Estimated GFR, mean (SD), mL/min/1.73 m^2^	4.31 (1.01)	4.83 (1.53)	0.38
Hemoglobin, mean (SD), g/dL	11.72 (1.69)	11.08 (1.06)	0.34
Calcium, mean (SD), mg/dL	9.48 (0.53)	9.48 (0.56)	0.97
Phosphorus, mean (SD), mg/dL	5.03 (0.86)	4.80 (0.93)	0.58
Parathyroid hormone, median (IQR), pg/mL	362.80 (213.60, 562.20)	143.70 (53.10, 373.00)	0.22
Serum albumin, mean (SD), g/dL	3.68 (0.39)	3.52 (0.41)	0.39
C-reactive protein, median (IQR), mg/dL	0.00 (0.00, 0.07)	0.31 (0.17, 0.48)	0.02
**Patient-Reported Outcomes**			
PFS-R ^f^ score, mean (SD)	6.05 (1.21)	5.32 (1.01)	0.18
EORTC QLQ-C30 ^g^ score, median (IQR)	41.62 (33.50, 44.83)	44.91 (39.87, 47.86)	0.19
Physical activity level ^h^, No. (%)			>0.99
High	3 (30)	2 (22)	
Moderate	6 (60)	5 (56)	
Low	1 (10)	2 (22)	
PSQI ^i^ score, median (IQR)	10.00 (9.00, 11.00)	10.00 (9.00, 12.00)	0.68
HADS ^j^			
Anxiety, median (IQR)	7.00 (6.00, 12.00)	3.00 (3.00, 6.00)	0.03
Depression, mean (SD)	7.30 (4.55)	6.33 (4.47)	0.65

Abbreviations: MAP, mean arterial pressure; CCI, Charlson Comorbidity Index; BMI, body mass index; Kt/V, weekly urea clearance normalized to the volume of distribution of urea; CCr, creatinine clearance; D/P, dialysate/plasma creatinine ratio; PFS-R, Piper Fatigue Scale-Revised; EORTC QLQ-C30, European Organization for Research and Treatment of Cancer Quality of Life Questionnaire-Core 30; PSQI, Pittsburgh Sleep Quality Index; HADS, Hospital Anxiety and Depression Scale; IPAQ-SF, International Physical Activity Questionnaire—Short Form; SD, standard deviation; IQR, interquartile range. ^a.^ *p*-values were calculated using independent-samples *t* tests for normally distributed continuous variables, Mann–Whitney *U* test for nonnormally distributed continuous variables, and Fisher’s exact tests for categorical variables. ^b.^ All statistical tests were 2-sided. Baseline *p* values are presented descriptively and were not used to assess the success of randomization. ^c.^ MAP = (systolic pressure + 2 × diastolic pressure) ÷ 3. ^d.^ Total Kt/V is the sum of residual kidney Kt/V and peritoneal Kt/V. ^e.^ Total CCr is the sum of residual kidney CCr and peritoneal Ccr. ^f.^ PFS-R scores range from 0 to 10, with higher scores indicating more severe fatigue. ^g.^ QLQ-C30 scores range from 0 to 100, with higher scores indicating better quality of life. ^h.^ Physical activity level derived from total weekly MET values using the IPAQ-SF classification. ^i.^ PSQI scores range from 0 to 21, with higher scores indicating poorer sleep quality. ^j.^ HADS scores range from 0 to 21 for each subscale (anxiety and depression); higher scores indicate more severe symptoms.

**Table 2:** Primary and secondary outcomes at week 12 by study group.

	Control Group (*n* = 10)	Moxibustion Group (*n* = 9)	Between-Group Estimate (95% CI)	*p* Value ^a^
**Fatigue Outcome**				
**PFS-R**, mean (SD)	4.50 (1.30)	3.41 (0.80)	−1.09 (−2.15, −0.03)	0.04
**PFS-R change from baseline**, mean (95% CI)	−1.55 (−2.80, −0.31)	−1.91 (−3.22, −0.61)	−0.36 (−2.03, 1.31)	0.65
**Fatigue Severity Categories**, No. (%)				
Mild	3 (30)	4 (44)		0.65
Moderate	7 (70)	5 (56)		
Severe	0 (0)	0 (0)		
**Dialysis Index**				
4-h D/P, mean (SD)	0.56 (0.08)	0.58 (0.12)	0.02 (−0.08, 0.12)	0.68
Total Kt/V, mean (SD)	2.10 (0.39)	2.14 (0.31)	0.04 (−0.30, 0.39)	0.80
Residual kidney Kt/V, median (IQR)	0.20 (0.00, 0.40)	0.40 (0.12, 0.82)	0.16 (−0.18, 0.68)	0.18
Peritoneal Kt/V, mean (SD)	1.82 (0.28)	1.64 (0.28)	−0.18 (−0.45, 0.09)	0.17
Total CCr, mean (SD), L/week	47.64 (8.06)	68.43 (19.89)	20.79 (6.39, 35.19)	0.007
Residual kidney CCr, mean (SD), L/week	10.14 (11.99)	25.14 (22.35)	15.00 (−2.10, 32.10)	0.08
Peritoneal CCr, mean (SD), L/week	37.50 (5.39)	43.29 (11.51)	5.79 (−2.76, 14.34)	0.17
**Patient-Reported Outcomes**				
EORTC QLQ-C30 score, median (IQR)	37.50 (34.79, 44.02)	40.85 (38.16, 45.81)	3.31 (−2.26, 8.80)	0.13
Physical Activity Level, No. (%)				
High	3 (30)	2 (22)		0.59
Moderate	4 (40)	6 (67)		
Low	3 (30)	1 (11)		
PSQI score, mean (SD)	12.40 (4.27)	8.89 (3.95)	−3.51 (−7.51, 0.49)	0.08
HADS				
Anxiety, median (IQR)	8.00 (7.00, 9.00)	5.00 (2.00, 7.00)	−2.00 (−7.00, 0.00)	0.04
Depression, median (IQR)	7.50 (5.00, 10.00)	4.00 (3.00, 7.00)	−2.00 (−6.00, 2.00)	0.28

^a.^ Between-group differences were calculated as the moxibustion group minus the control group. For approximately normally distributed continuous outcomes, estimates are mean differences with 95% CIs, and *p*-values were calculated using independent-samples *t* tests. For nonnormally distributed continuous outcomes, estimates are Hodges–Lehmann location-shift estimates with 95% CIs, and *p*-values were calculated using the Mann–Whitney *U* tests. Categorical outcomes were compared using Fisher’s exact test. All tests were 2-sided. There was no adjustment for multiple comparisons because these analyses were exploratory.

**Table 3:** Adverse events during the 12-week intervention period.

	Control Group (*n* = 10)	Moxibustion Group (*n* = 9)	*p* Value ^a^
**Participants with ≥1 AE, No. (%)**	2 (20.0%)	4 (44.4%)	0.35
**Total number of AEs**	4	7	-
**Intervention-related AEs**			
Constipation	0	1	-
**AEs unrelated to intervention**			
Hypotension	1	1	-
Infections ^b^	3	3	-
Other ^c^	0	2	-

^a.^ The *p* value compares the proportions of those participants experiencing at least one adverse event using Fisher’s exact test. ^b.^ Infections included upper respiratory tract infection, urinary tract infection, and folliculitis. ^c.^ Other adverse events included dialysate leakage and non-menstrual vaginal bleeding.

## Data Availability

Due to patient confidentiality concerns, data are only available upon reasonable request.

## Supplementary Materials

The following supporting information can be downloaded at: https://www.mdpi.com/article/10.3390/healthcare14183110/s1, Supplement S1: Study Protocol [19,20,41,42,43,44,45,46]; Supplement S2: Supplemental Online Content: Figure S1: Structure of the Moxibustion Device Used in Home-Based Intervention; Figure S2: Demonstration of Moxibustion Application at the Shenque (CV8) Acupoint; Figure S3: Changes in Patient-Reported Outcomes Over Time by Group; Table S1: Study Inclusion and Exclusion Criteria; Table S2: Primary and Secondary Outcomes; Table S3: Nutritional and Laboratory Outcomes at Week 12 by Study Group; Table S4: Between-Group Comparisons of PFS–R Total and Domain Scores, and Fatigue Severity Categories at each Time point; Table S5: Between-Group Comparisons of Patient-Reported Outcomes at each Time point; Table S6: Within-Group Estimated Changes From Baseline to Week 12 in Secondary Outcomes; Table S7: Between-Group Differences in Change From Baseline to Week 12 in Secondary Outcomes; Table S8: Repeated-Measures ANOVA for Time, Group, and Interaction Effects on Primary and Patient-Reported Outcomes.
