# Supplementary material for: The Feasibility of Home-Based Moxibustion for Fatigue in Peritoneal Dialysis: A Randomized Controlled Pilot Trial
Source: Healthcare (Basel). 2026 Sep 20;14(18):3110. doi: 10.3390/healthcare14183110 (PMC13606285; doi:10.3390/healthcare14183110)
Supplement: Supplementary file 1 [file healthcare-14-03110-s001.zip › Supplement1Study Protocol-clean 1.pdf]

Supplemental 1. Study Protocol

Home-Based Moxibustion for Fatigue in Peritoneal Dialysis: A Randomized Pilot Trial

Funding: The Science and Technology Research Fund from Guangdong provincial hospital of Chinese medicine (No.YN2020QN24)

Principal Investigators:
La Zhang, PhD, M.Med.

Co-Investigators:
Fuhua Lu, MD

Study site: Guangdong Provincial Hospital of Chinese Medicine

Final Approved Version: October 17, 2022/version 3

Summary of Protocol Changes

This study protocol was initially approved by the Ethics Committee of Guangdong Provincial Hospital of Chinese Medicine and registered in the Chinese Clinical Trial Registry (ChiCTR2300070682). During the conduct of the trial, minor but important methodological refinements were made to improve analytic rigor and clarity. These modifications are summarized as follows:

1. Specification of Statistical Analyses:
The protocol was amended to provide more detailed descriptions of the statistical methods for both primary and secondary outcomes.

- The primary outcome—the between-group difference in Piper Fatigue Scale (PFS) scores at week 12—was analyzed using independent-sample t tests and repeated-measures analysis of variance (ANOVA). Fatigue severity categories were compared using chi-square (χ^2) tests.
- Secondary outcomes were analyzed using t tests or Wilcoxon rank-sum tests for continuous variables and χ^2 tests or Fisher exact tests for categorical variables, depending on distribution.
- Within-group changes from baseline were analyzed using paired t tests, and post hoc analyses assessed between-group differences in change-from-baseline scores for selected endpoints.

2. Handling of Missing Data:
The protocol was updated to specify that missing data would be addressed using multiple imputation via chained equations, consistent with best practices for handling incomplete outcome data.

All modifications were reviewed and approved by the study's statistical and methodological team. These changes did not alter the primary aims of the trial and were made prior to unblinding and data analysis to maintain analytic transparency and integrity.

Table of Contents

1. Scheme Summary ... 3
2. Background and Rationale ... 4
3. Objectives ... 5
4. Study Design ... 5
5. Participants ... 5
    5.1 Inclusion criteria ... 5
    5.2 Exclusion criteria ... 5
    5.3 Criteria for exclusion from analysis ... 6
    5.4 Criteria for dropout ... 6
    5.5 Criter for study termination ... 6
6. Sample size and Randomization ... 6
    6.1 Sample size estimation ... 6
    6.2 Randomization ... 6
7. Interventions ... 6
8. Study Procedures ... 8
    8.1 Study timeline diagram ... 8
    8.2 Screening and recruitment ... 9
    8.3 Run-In Phase (Week –1 to 0) ... 9
    8.4 Intervention Phase (Week 0 to 12) ... 9
    8.5 Schedule of enrollment, interventions, and assessments ... 10
9. Outcome Measure ... 12
    9.1 Primary outcome ... 12
    9.2 Secondary outcome ... 12
10. Safety Monitoring ... 12
    10.1Adverse Events ... 12
        10.1.1 Definition ... 12
        10.1.2 Observation and documentation ... 12
        10.1.3 Assessment of causality ... 13
        10.1.4 Assessment of Severity ... 13
        10.1.5 Adverse Events of Special Interest (AESIs) ... 13
    10.2 Serious Adverse Events (SAEs) ... 13
        10.2.1 Definition ... 13
        10.2.2 Reporting of serious adverse events ... 13
11. Risk Evaluation and Management Plan ... 13
    11.1 Local skin reactions to moxibustion ... 13
    11.2 Moxibustion-induced dizziness ... 14
    11.3 Common complications of PD[5-8] ... 14
        11.3.1 Abnormal drainage ... 14
        11.3.2 Catheter exit site or tunnel infections ... 14
        11.3.3 Contamination during exchange ... 14
        11.3.4 Dialysate leak ... 14
        11.3.5 Leaking or cracked transfer set ... 14

11.3.6 Infusion or drainage failure 15
11.3.7 Catheter rupture 15
11.3.8 Discomfort or pain during infusion 15
12. Data Management and Statistical Analysis 15
12.1 Data management and quality control 15
12.2 Statistical Analysis Plan 15
13. Participant Privacy and Confidentiality 15
14. Ethics 15
Reference 16

1. Scheme Summary

Study Title	Home-Based Moxibustion for Fatigue in Peritoneal Dialysis: A Randomized Pilot Trial
Study Objective	(1) To preliminarily assess the efficacy and safety of home-based moxibustion in relieving fatigue symptoms in patients undergoing peritoneal dialysis;<br>(2) To explore the feasibility and adherence of implementing moxibustion as a self-administered therapy in the peritoneal dialysis population.
Design	A single-center, prospective, randomized controlled pilot trial.
Intervention Group	Moxibustion group (10 participants)
Control Group	Wait-list control group (10 participants)
Sample size	20
Inclusion criteria	1. Adults aged 18 years or older;<br>2. Receiving maintenance peritoneal dialysis for at least 4 months with clinically stable condition prior to enrollment;<br>3. Reporting persistent fatigue symptoms for ≥ 4 weeks and a total score of ≥ 4 on the Piper Fatigue Scale;<br>4. Capable of verbal communication, without cognitive or language impairment;<br>5. Willing to participate in the study and able to provide written informed consent.
Exclusion criteria	1. Life expectancy less than 6 months;<br>2. Receiving concurrent hemodialysis or with prior kidney transplantation;<br>3. Experiencing severe anemia, infection acute exacerbation of heart failure, or other comorbidities requiring recent adjustment of peritoneal dialysis regimen;<br>4. Having impaired nutritional intake or moderate-severe malnutrition (defined as serum albumin < 25g/L);<br>5. Presence of local skin conditions, (e.g., lesions, rashes, ecchymosis, or petechiae) that may contraindicate moxibustion;<br>6. History of fatigue-targeted therapies or changes to the dialysis protocol for fatigue relief within 4 weeks prior to enrollment;<br>7. Previous hypersensitivity or serious adverse reaction to moxibustion, or other reasons precluding adherence to the intervention protocol;<br>8. Co-existing medical conditions unrealted to end-stage renal disease that may

	contribute to fatigue (e.g., malignancy or other chronic wasting illnesses);<br>9. Concurrent participation in another clinical trial.
Feasibility and Efficacy Outcome	Primary Outcomes:<br>1.Between-group difference in mean scores on the Piper Fatigue Scale (PFS) at week 12.<br>2.Proportion of participants in the moxibustion group who achieved the target therapeutic dose (defined as completing home-based moxibustion at least 3 times per week for 12 weeks).<br>Secondary Outcomes:<br>1. Between-group differences at week 12 in laboratory indicators related to peritoneal dialysis adequacy and metabolic status, including serum creatinine, blood urea nitrogen, serum albumin, hemoglobin, calcium, phosphorus, parathyroid hormone, weekly Kt/V, and total creatinine clearance.<br>2. Between-group differences in scores on the European Organization for Research and Treatment of Cancer Quality of Life Questionnaire Core 30 (EORTC QLQ-C30).<br>3. Between-group differences in sleep quality measured by the Pittsburgh Sleep Quality Index (PSQI).<br>4. Between-group differences in psychological distress measured by the Hospital Anxiety and Depression Scale (HADS).<br>5. Between-group differences in physical activity level assessed by the International Physical Activity Questionnaire–Short Form (IPAQ-SF).
Safety Outcome	1.Incidence of local skin burns related to moxibustion.<br>2.Incidence of adverse events during the intervention period.

2. Background and Rationale

Peritoneal dialysis (PD) is one of the most commonlu used modalities of renal replacement therapy for patients with end-stage kidney disease (ESKD). Compared with hemodialysis, PD offered greater flexibility regarding treatment location, better preservation of residual kidney function, and fewer diatary restrictions, facilitating patients' reintegration into daily and social life. Impotantly, PD can be perfromed at home, reducing the need for frequent visits to healthcare facilities and significantly lowering healthcare costs and ;abor demands. As the number of patients with ESKD continues to rise, PD has become a preferred and policy-supported dailys modality, particularly in regions with limited healthcare resources.

Despite its advantages, PD patients continue to experience substantial symptom burdens, including infections, physical discomfort, and fatigue. Among these, fatigue is not only the most common but also one of the most distressing symptoms, consistently identified as a top clinical concern across PD polulations worldwide. Fatigue in PD patients typically manifests as persistent exhaustion or lack of energy, while severely impairs quality of life and daily functioning. Studies reported that 60% to 97% of long-term dialysis patients suffered from fatigue. While anemia, inadequate clearance of uremic toxins, malnutrition, and insomnia may contribute to fatigue, many patients experience persistent symptoms even after these underlying factors have been addressed. Chronic fatigue was associated with worsened psychological, social, and physical health outcomes and was closely linked to poor

prognosis. Therefore, developing safe and effective interventions to alleviate fatigue in PD patients is of significant clinical importance.

Moxibustion is a traditional Chinese medicine therapy that applies thermal stimulation generated by bruning mugwort (*Artemisia argyi*) at specific acupoints and meridians to restore yin-yang balance in the body. It is widely accepted among patients due to its simplicity, low cost, and suitability for long-term use. Several small-scale clinical trials have suggested that moxibustion may effectively relieve fatigue associated with sub-health conditions or various diseases, thereby improving patients' quality of life. However, no clinical evidence currently exists regarding the use of moxibustion for fatigue in PD patients. The feasibility, efficacy, and safety of this approach in PD poluations remained to be established.

3. Objectives

This study aims to evaluate the feasibility and adherence of home-based moxibustion therapy among patients undergoing PD, and to preliminarily assessed its efficacy and safety in alleviating fatigue in these populations.

The findings are intended to inform the development of a practical, traditional Chinese medicine-based approach for fatigue management in PD populations, and to support the broader integration of moxibustion as a culturally adapted adjunctive therapy.

4. Study Design

This study is designed as a prospective, randomized, controlled pilot trial.

5. Participants

5.1 Inclusion criteria

Participants will be eligible for enrollment if they met all of the following criteria:

(1) Adults aged 18 years or older;

(2) Receiving maintenance peritoneal dialysis for at least 4 months with clinically stable condition prior to enrollment;

(3) Reporting persistent fatigue symptoms for ≥ 4 weeks and a total score of ≥ 4 on the Piper Fatigue Scale;

(4) Capable of verbal communication, without cognitive or language impairment;

(5) Willing to participate in the study and able to provide written informed consent.

5.2 Exclusion criteria

Participants will be excluded if they met any of the following criteria:

(1) Life expectancy less than 6 months;

(2) Receiving concurrent hemodialysis or with prior kidney transplantation;

(3) Experiencing severe anemia, infection acute exacerbation of heart failure, or other comorbidities requiring recent adjustment of peritoneal dialysis regimen;

(4) Having impaired nutritional intake or moderate-severe malnutrition (defined as serum albumin < 25g/L);

(5) Presence of local skin conditions, (e.g., lesions, rashes, ecchymosis, or petechiae) that may contraindicate moxibustion;

(6) History of fatigue-targeted therapies or changes to the dialysis protocol for fatigue relief within 4

weeks prior to enrollment;

(7) Previous hypersensitivity or serious adverse reaction to moxibustion, or other reasons precluding adherence to the intervention protocol;

(8) Co-existing medical conditions unrealted to end-stage renal disease that may contribute to fatigue (e.g., malignancy or other chronic wasting illnesses);

(9) Concurrent participation in another clinical trial.

5.3 Criteria for exclusion from analysis

Participants will be excluded from the final analysis set if they met either of the following conditions:

(1) Inadvertent enrollment of individuals who fail to meet the eligibility criteria;

(2) Missing baseline data or failure to complete the minimum moxibustion exposure, defined as fewer than three 15-minute home-based sessions per week during the intervention period.

5.4 Criteria for dropout

Participants will be considered to have dropped out if:

(1) The participant voluntarily withdraws informed consent or discontinued participant for personal reasons;

(2) The participant misses scheduled visits and could not be be contacted despite repeated attempts.

5.5 Criter for study termination

Participants will be withdrawn from the study under the following circumstances:

(1) Occurrence of serious cardiovascular or cerebrovascular events, surgical emergencies, or other critical illnesses affecting major organ systems during the follow-up period;

(2) Occurrence of serious adverse events;

(3) Development of severe infection or skin injury (e.g., dizziness or local suppuration) related to improper moxibustion technique.

6. Sample size and Randomization

6.1 Sample size estimation

This study is designed as a superiority trial. The primary outcome is the between-group difference in mean scores on the Piper Fatigue Scale (PFS) at week 12. According to previous study [1], the mean (standard deviation, SD) post-intervention PFS scores were 3.77 ± 0.50 in the intervention group and 4.88 ± 0.61 in the blank control group. Using a two-sided significance level of 5% and a statistical power of 90%, the required sample size was calculated to be 8 participants per group. Assuming a 20% dropout rate, a total of 18 participants would be needed. In alignment with expert recommendations by Professor Jianping Liu from the Beijing University of Chinese Medicine on determining sample size for pilot studies in traditional Chinese medicine clinical research [2], we planned to enroll approximately 18 participants in this pilot trial to better assess the feasibility of the study protocol.

6.2 Randomization

A computer-generated random sequence is used to assign participants to study groups via a centralized web-based randomization system. After obtaining informed consent, eligible participants will be allocated to either the intervention or control group according to the randomization result. The random sequence and group assignment information are managed by a designated team member who will not be involved in participant enrollment or follow-up.

7. Interventions

All participants receive one in-person training session and an instructional booklet on managing daily

life to alleviate dialysis-related fatigue. Participants in the moxibustion group are trained to perform home-based moxibustion prior to the intervention, while participants in the control group are offered the same training and equipment after study completion at week 12.

To ensure standardized and safe application of home-based moxibustion, training is conducted during the run-in period in the reception room of the Peritoneal Dialysis Center at the hospital by trained physicians. Participants are guided through the full moxibustion procedure during the session.

The training includes the following components:

(1) Preparation: Participants are advised to perform moxibustion in a warm, quiet room, preferably during peritoneal dialysate dwell time. Required materials include a moxibustion device, moxa cones, and a lighter. The recommended posture is supine position.

(2) Operation procedure:

- Open the moxibustion device by rotating the body and lid. Insert the moxa cone onto the internal pin, ignite it, align and secure the lid by tightening the rotating mechanism.
- Adjust the height of the moxa cone to regulate heat intensity using the temperature control feature. Then, secure the device over the umbilicus (Shenque acupoint, CV8) using the elastic strap. Moxibustion is performed until a comfortable warming sensation is felt, and local skin becomes mildly flushed (Figure 1).
- Remove the device when the moxa cone has fully burned or after 15 minutes of treatment.

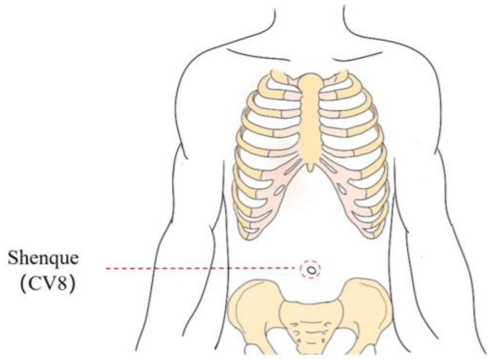

Figure 1. Anatomical location of acupoint CV8 (Shenque).

(3) Temperature monitoring: Participants are assisted in adjusting the preset temperature to a tolerable level using an infrared thermometer. The maximum allowable surface temperature is limited to 43°C.

(4) Safety education: Participants are educated about potential risks of home-based moxibustion and how to address common issues.

(5) Competency check: Participants must demonstrate proper procedure and pass an operating assessment before entering the study.

7.1 Moxibustion group

Participants in the moxibustion group perform home-based therapy using a standardized device made of carbonized bamboo and a built-in iron mesh to prevent ash from contacting the skin (Figure 2). Lit moxa cones are inserted into the cylindrical device and positioned over the Shenque acupoint (CV8). Each session lasts at least 15 minutes and is performed at least 3 times per week. Treatment is self-limited based on the participant’s perception of warmth and the presence of mild skin flushing at

the application site.

7.2 Control group

Participants in the control group do not receive any acupuncture, pharmacological, or physical therapy specifically aimed at relieving fatigue symptoms during the 12-week trial period. After completing the study at week 12, they are offered compensatory moxibustion treatment, including standardized training on moxibustion procedures and provision of moxa core and moxibustion devices, in accordance with the intervention protocol used in the moxibustion group.

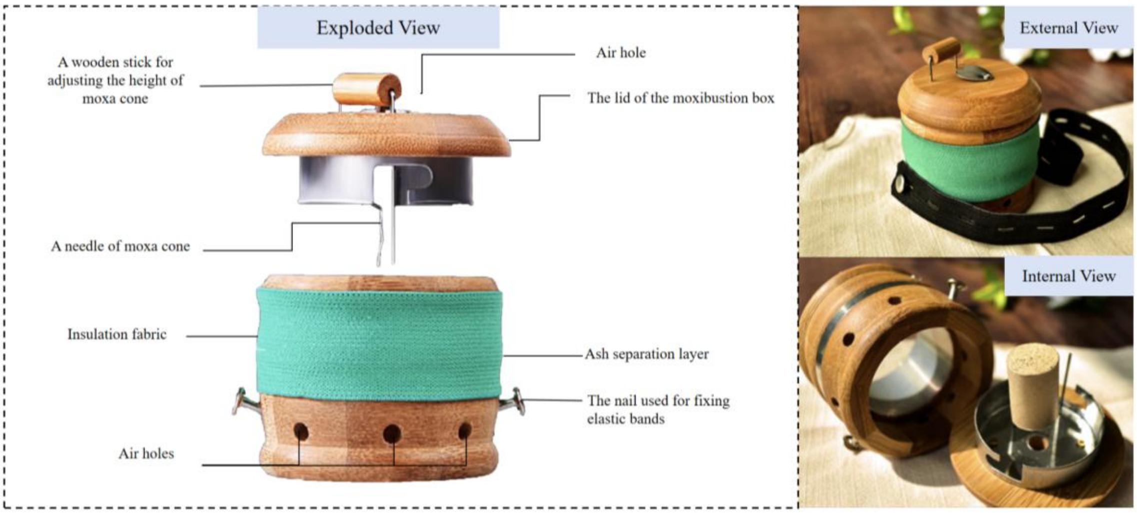

Figue2. Illustration of Moxibustion Devices

8. Study Procedures

8.1 Study timeline diagram

This diagram summarizes the procedural steps and data collection timepoints across the study （Figure 3).

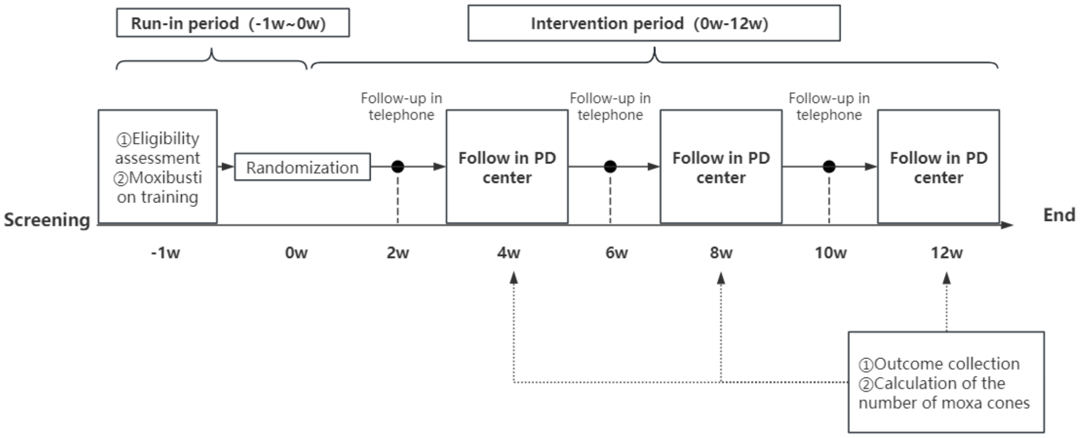

Figure 3. Study Timeline Diagram

8.2 Screening and recruitment

Eligible PD patients are recruited from the PD outpatient clinic and in-patient wards of the Department of Nephrology at Guangdong Provincial Hospital of Chinese Medicine. The study team first provides patients with an overview of the trial, including its objectives, intervention procedures, and research personnel. If the patient expresses interest, the investigator collects baseline data (e.g., medical history and demographic information) in a designated consultation room and administers the Piper Fatigue Scale (PFS). Patients who meet the inclusion criteria based on PFS scores and other criteria are formally enrolled after providing written informed consent.

8.3 Run-In Phase (Week –1 to 0)

Participants are randomly assigned in a 1:1 ratio to the moxibustion group or the wait-list control group using a centralized computerized randomization system. Within one week of allocation, participants in the moxibustion group are scheduled for standardized training on home-based moxibustion procedures in a designated training room. They are also instructed on how to complete the patient diary and are briefed on relevant study instructions. Only participants who pass the skills assessment receive the moxibustion device and supplies.

During this phase, baseline laboratory assessments are completed, including complete blood count, renal function panel (4 items), liver function tests (12 items), electrolytes level (6 items), parathyroid hormone, dialysis adequancy and peritoneal equilibration test. Participants also complete baseline patient-reported outcome measures, including the EORTC QLQ-C30, Pittsburgh Sleep Quality Index (PSQI), Hospital Anxiety and Depression Scale (HADS), and the International Physical Activity Questionnaire–Short Form (IPAQ-SF).

8.4 Intervention Phase (Week 0 to 12)

(1) First Telephone Follow-Up (Week 2): Participants are contacted to assess adherence to home-based moxibustion, use of moxa cones, and the presence of any adverse effects such as blistering. Investigators provide consultation and remind participants of the upcoming in-person visit.

(2) First In-Person Visit (Week 4): Changes in fatigue symptoms are evaluated and the PFS and other relevant questionnaires are administered. Investigators inquire about any discomfort or adverse effects experienced during treatment (If appropriate). Participants return their diary and unused moxa cones with packaging. A four-week supply of new moxa cones is provided, and the next visit is scheduled.

(3) Second Telephone Follow-Up (Week 6): A follow-up call is made to assess continued adherence, use of moxa cones, and any adverse event. Investigators provide consultation and a reminder for the next in-person visit.

(4) Second In-Person Visit (Week 8): Fatigue symptoms are reassessed using the PFS and other scales. Adverse events are documented (if appropriate). Participants return diary and unused moxa cones with packaging. Another four-week supply is distributed and the final visit is scheduled.

(5) Third Telephone Follow-Up (Week 10): A follow-up call is made to assess continued adherence, use of moxa cones, and any adverse event. Investigators provide consultation and a reminder for the end-of-study visit.

(6) Final In-Person Visit (Week 12): Final assessments are conducted, including the PFS and related scales. Participants return diary and remaining moxa cones. Blood and peritoneal dialysate samples are collected for follow-up laboratory testing (including CBC, renal function panel, liver function panel, electrolytes, PTH, dialysis adequancy and PET). At this visit, participants in the wait-list control group are provided with compensatory moxibustion training and supplies, following the same procedures used for the moxibustion group.

8.5 Schedule of enrollment, interventions, and assessments

This table presents the timeline of study procedures across baseline and weeks 2 to 12, including screening, intervention delivery, treatment monitoring, and outcome assessments.

Table 1. Schedule of enrollment, interventions, and assessments

Timepoint	Screening	Baseline (week 0)	week 2	week 4	week 6	week 8	week 10	week 12
Visits	1	2	3	4	5	6	7	8
Informed Consent	√							
Eligibility Assessment	√							
Randomization		√						
Moxibustion Training (Intervention Group only)	√	√						
Distribution of moxibustion supplies (Intervention Group only)		√		√		√		
Medical History Ccollection		√						
Vital Signs and Physical Exam	√	√		√		√		√
Labotory tests*		√						√
Dialysis adequancy and peritoneal equibillium tests		√						√
PFS-R	√	√		√		√		√
QLQ-C30		√		√		√		√
PSQI		√		√		√		√
IPAQ-SF		√		√		√		√
HADS		√		√		√		√
Adverse Events Collection			√	√	√	√	√	√
Patient Diary Review				√		√		√
TelePhone Follow-up			√		√		√	
In-site Follow-up				√		√		√

Abbreviation: PFS-R, Piper Fatigue Scale–Revised; EORTC QLQ-C30, European Organization for Research and Treatment of Cancer Quality of Life Questionnaire-Core 30; PSQI, Pittsburgh Sleep Quality Index; HADS, Hospital Anxiety and Depression Scale; IPAQ-SF, International Physical Activity Questionnaire–Short Form.

* Labotory tests include: Complete Blood Count，Serum Electrolyte Panel, Serum creatinine (SCr), Blood urea nitrogen (BUN), eGFR, Intact Parathyroid Hormone and Liver Function Tests.

PFS indicates the revised Piper Fatigue Scale; QLQ-C30, the European Organization for Research and Treatment of Cancer Quality of Life Questionnaire; PSQI, Pittsburgh Sleep Quality Index; HADS, Hospital Anxiety and Depression Scale; IPAQ-SF, International Physical Activity Questionnaire–Short Form.

9. Outcome Measure

The following outcome measures will be assessed by the participants themselves or the by dependent evaluators.

9.1 Primary outcome

The primary outcome will be the between-group difference in mean total scores on the Piper Fatigue Scale (PFS) at the end of the 12-week intervention. The PFS is a validated instrument commonly used in clinical studies to measure fatigue severity. It comprises 22 items and typically requires about 2 minutes to complete. Originally developed for assessing cancer-related fatigue, the PFS has also been validated in other populations, including patients undergoing dialysis [1,3,4]. It evaluates fatigue across four dimensions: sensory, cognitive/mood, behavioral, and affective meaning. Total scores range from 0 to 10, with 0 indicating no fatigue, 1–3 indicating mild fatigue, 4–6 moderate fatigue, and 7–10 severe fatigue.

In addition, feasibility and adherence will be evaluated as co-primary outcomes. The proportion of participants who complete the full intervention—defined as at least three moxibustion sessions per week for 12 consecutive weeks—will be calculated to assess protocol adherence and feasibility.

9.2 Secondary outcome

(1) Biochemical markers: Between-group differences in the change from baseline to week 12 will be assessed for serum creatinine, blood urea nitrogen, serum albumin, hemoglobin, serum calcium, serum phosphorus, and intact parathyroid hormone.

(2) Dialysis adequacy: Weekly Kt/V, total creatinine clearance and peritoneal equilibrium tests at week 12, will be compared between groups.

(3) Patient-reported outcomes (PROs): Between-group differences in changes in scores from baseline to week 12 will be assessed using the following validated instruments:

- European Organization for Research and Treatment of Cancer Quality of Life Questionnaire-Core 30 (EORTC QLQ-C30)
- Pittsburgh Sleep Quality Index (PSQI)
- Hospital Anxiety and Depression Scale (HADS)
- International Physical Activity Questionnaire–Short Form (IPAQ-SF)

10. Safety Monitoring

10.1Adverse Events

10.1.1 Definition

An adverse event (AE) will be defined as any unfavorable medical occurrence in a participant after receiving the trial intervention. This may include symptoms, clinical signs, incident diseases, or abnormal laboratory results, regardless of whether there is a clear causal relationship with the intervention.

10.1.2 Observation and documentation

All adverse events occurring during the study, including abnormal laboratory findings, will be actively monitored and documented. Investigators will assess the nature, severity, and causal

relationship to the intervention for each adverse event, and will record the findings in the Case Report Form (CRF) in a timely and accurate manner.

10.1.3 Assessment of causality

The relationship between the adverse event and the intervention will be categorized as:

- Definitely related
- Probably related
- Possibly related
- Possibly unrelated
- Definitely unrelated

For statistical analysis of adverse reaction rates, only events classified as "definitely related," "probably related," or "possibly related" will be included.

10.1.4 Assessment of Severity

Adverse events will be graded according to the following criteria:

- Mild: Transient symptoms that do not interfere with daily activities
- Moderate: Noticeable discomfort that affects daily activities; tolerable without terminating the intervention
- Severe: Significant symptoms that interfere with or prevent daily activities; intolerable and require discontinuation of the intervention

10.1.5 Adverse Events of Special Interest (AESIs)

Participants will be specifically monitored for adverse events of special interest (AESIs) related to the application of home-based moxibustion therapy. These include, but are not limited to:

- Local skin reactions, such as burns, redness, blistering, or pigmentation at the moxibustion site
- Moxibustion-related dizziness or syncope, possibly caused by heat sensitivity or prolonged prone positioning
- Allergic reactions to moxa smoke or herbal components
- Respiratory discomfort or other symptoms caused by smoke inhalation in poorly ventilated environments

Participants will be instructed to report these symptoms promptly during scheduled visits or telephone follow-ups. Investigators will document the incidence, severity, and resolution of each event, and determine whether trial continuation is appropriate based on clinical judgment.

10.2 Serious Adverse Events (SAEs)

10.2.1 Definition

A serious adverse event (SAE) will be defined as any adverse medical occurrence that results in death, is life-threatening, causes permanent or significant disability or incapacity, requires hospitalization or prolongation of existing hospitalization, or results in a congenital anomaly or birth defect.

10.2.2 Reporting of serious adverse events

In the event of an SAE, the investigator will be required to report it to the principal investigator within 24 hours and will complete the Serious Adverse Event Reporting Form.

11. Risk Evaluation and Management Plan

11.1 Local skin reactions to moxibustion

- Localized erythema and warmth are commonly observed following moxibustion and

typically resolve spontaneously without the need for treatment.

- If superficial skin burns occur, mild edema or small blisters (diameter ≤ 1 cm) may develop. These generally require no intervention and can self-resolve. For larger blisters, a sterile needle may be used to puncture and drain the fluid. The blister can be trimmed to expose the underlying damaged layer, and topical antibiotic ointments may be applied to prevent infection. If sterile exudate forms, it is typically not removed. The lesion usually heals within 5–8 days without scarring.
- In cases where deeper dermal tissue is damaged, localized swelling, erosion, or sterile suppuration may occur. Minor injuries often heal with shallow scarring within 7–20 days. More extensive injuries may require 20–50 days to crust and heal and may result in permanent scarring.
- During the suppurative phase, physical activity should be limited, and infection should be avoided. If local infection occurs, mild erythema or redness can be managed with topical anti-inflammatory agents. In more severe cases with pain, heat, or spreading inflammation, oral or topical antibiotics may be necessary. Deep infections in skin should be treated in a medical facility.

11.2 Moxibustion-induced dizziness

If dizziness or fainting occurs during moxibustion, the session should be stopped immediately. The patient should lie flat, remain warm, and rest. In mild cases, symptoms often resolve with rest. Severe episodes should be treated as syncope, and the patient should seek emergency medical care.

11.3 Common complications of PD [5-8]

11.3.1 Abnormal drainage

- Cloudy peritoneal effluent: Retain the effluent and immediately contact the dialysis center. Bring the sample to the hospital for evaluation and treatment.
- Increased fibrin in effluent: Small amounts may be normal; large amounts may suggest catheter obstruction. Patients should contact the dialysis center.
- Hemorrhagic effluent: If the color is light pink and the amount is minimal, then keep observation. If heavy bleeding is suspected, use a new cold bag of dialysate and observe changes. If bleeding persists, contact the dialysis center.

11.3.2 Catheter exit site or tunnel infections

Manifested as redness, polyps, swelling, tenderness, or discharge. Increase the frequency of dressing changes and apply mupirocin ointment. If symptoms persist, contact the dialysis center promptly.

11.3.3 Contamination during exchange

- If the dialysate or tubing is touched during exchange, immediately stop the procedure.
- If the dialysate bag's connector is contaminated, discard and replace the bag.
- If the transfer set is contaminated, clamp the tubing, replace the iodine cap, and contact the dialysis center for a new transfer set.

11.3.4 Dialysate leak

If leakage is detected before the exchange, discard the dialysate and use a new bag. If leakage occurs during the procedure, stop dialysis, clamp the tubing near the abdomen, and notify the dialysis center.

11.3.5 Leaking or cracked transfer set

Clamp the tubing near the site of leakage or rupture and contact the dialysis center. Do not reuse the damaged set before replacement.

11.3.6 Infusion or drainage failure

Check that all clamps and valves are open. Ensure tubing is not kinked or twisted.Change body position and observe for improvement.Assess for constipation, which may cause catheter malposition and impede drainage. Use laxatives if needed under medical guidance.

If fibrin clots or precipitates are suspected, contact the dialysis team immediately.

11.3.7 Catheter rupture

If the external catheter detaches from the titanium connector or if the catheter is torn, clamp the tubing close to the abdomen, wrap with sterile gauze, and contact the dialysis center immediately for emergency management.

11.3.8 Discomfort or pain during infusion

Ensure appropriate dialysate temperature and reduce the height of the dialysate bag to slow the infusion rate. Check for abnormalities in the prior drainage bag.

12. Data Management and Statistical Analysis

12.1 Data management and quality control

To ensure the quality of the study, all personnel involved must complete training related to clinical research. Standard operating procedures have been established, and uniform training has been provided to all participants. The study is conducted strictly in accordance with these standardized procedures. Randomization was performed by the methodology team. Designated members of the study team regularly monitor the clinical trial to verify adherence to the trial protocol and to ensure that case report forms are accurately and thoroughly completed.

12.2 Statistical Analysis Plan

Statistical analyses will be performed independently by statisticians blinded to group assignments, using Stata 12.0 (StataCorp) or SPSS version 19.0 (IBM Corp). All tests will be two-sided, and a P value of <.05 will be considered statistically significant. Descriptive statistics will be used to summarize demographic characteristics and baseline values. Continuous variables will be expressed as mean ± standard deviation; medians and 95% confidence intervals will be reported when appropriate. Between-group differences will be analyzed using analysis of variance (ANOVA) or the Kruskal–Wallis test, depending on data distribution. Categorical variables will be presented as counts and percentages and will be compared using the chi-square test or Fisher exact test, as appropriate.

13. Participant Privacy and Confidentiality

The results of this study may be published in medical journals; however, all patient information will be kept strictly confidential in accordance with legal and ethical requirements. Personal identifiers will not be disclosed unless required by applicable laws and regulations. Access to participants' original records may be granted to authorized personnel from regulatory authorities, the hospital ethics committee, or designated oversight bodies, as needed and in compliance with relevant procedures.

14. Ethics

This study will be conducted in accordance with the Declaration of Helsinki and relevant local

regulations.

This trial protocol, along with the case report forms and informed consent documents, will be submitted for written approval by the Ethics Committee of Guangdong Provincial Hospital of Chinese Medicine prior to study initiation.

The investigator or a designated research team member will be responsible for explaining the risks and potential benefits of participation to each patient, the patient's legally authorized representative, or an impartial witness. Written informed consent will be obtained before any study-related procedures are conducted.

Original, signed, and dated informed consent forms, co-signed by the participant (or their legal representative) and the person conducting the consent process, will be retained by the investigator in accordance with regulatory and institutional requirements.

Reference

1. Huang, A.; Zhang, S.; Song, F. The Influence of Aerobic Exercise on Fatigue Status in Peritoneal Dialysis Patients. Jilin Med. J. 2012, 33, 1149–1150. https://doi.org/10.3969/j.issn.1004-0412.2012.06.023.

2. Zhang, Y, Wang, J, Hu, Y, Shao, B, Fu, Z, Liu, J. Determining Sample Size of Pilot Trials in Traditional Chinese Medicine. J. Tradit. Chin. Med. 2021;62(04):307-311. doi:10.13288/j.11-2166/r.2021.04.008

3.Piper, B.F.; Dibble, S.L.; Dodd, M.J.; Weiss, M.C.; Slaughter, R.E.; Paul, S.M. The revised Piper Fatigue Scale: Psychometric evaluation in women with breast cancer. Oncol. Nurs. Forum 1998, 25, 677–684.

4.Kim, K.H.; Lee, M.S.; Kim, T.H.; Kang, J.W.; Choi, T.Y.; Lee, J.D. Acupuncture and related interventions for symptoms of chronic kidney disease. Cochrane Database Syst. Rev. 2016, 2016, Cd009440. https://doi.org/10.1002/14651858.CD009440.pub2

5. Chen, X. Peritoneal Dialysis Standard Operating Procedure (SOP); People's Military Medical Press: Beijing, China, 2010.

6. Project Group of "White paper on the status of peritoneal dialysis management in China". White paper on the current status of peritoneal dialysis management in China. Chin. J. Nephrol. 2022, 38, 1076-1104. https://doi.org/10.3760/cma.j.cn441217-20220418-00158

7. Li, P.K.T., Chow, K.M., Cho, Y., Fan, S., Figueiredo, A.E., Harris, T., Kanjanabuch, T., Kim, Y.L., Madero, M., Malyszko, J.; et al. ISPD peritonitis guideline recommendations: 2022 update on prevention and treatment. Perit. Dial. Int. 2022, 42, 110-153. https://doi.org/10.1177/08968608221080586

8.Morelle, J.; Stachowska-Pietka, J.; Öberg, C.; Gadola, L.; La Milia, V.; Yu, Z.; Lambie, M.; Mehrotra, R.; de Arteaga, J.; Davies, S. ISPD recommendations for the evaluation of peritoneal membrane dysfunction in adults: Classification, measurement, interpretation and rationale for intervention. Perit. Dial. Int. 2021, 41, 352-372. https://doi.org/10.1177/0896860820982218

Appendix 1. Piper Fatigue Scale License Certificate

Thank you for your order. Your transaction has been completed, and a receipt for your purchase has been emailed to you from Paypal. For reference your order number is 5Y755955XM280203J.

Please use the links below to download your purchase.

To download Piper Fatigue Scale (PFS) click the link below:
Piper Fatigue Scale (PFS) FULL.pdf (200 KB) ~ Download link will expire in 2 days

These links have also been emailed to you at zheheng.qiu@gmail.com.

If you have any questions regarding this order please contact us.

AgedCareTests
https://www.agedcaretests.com
info@agedcaretests.com

Appendix 2. Informed Consent Form: Signature Page Template

知情同意书 版本号：3.0/20221017

知情同意书・同意签字页

Signature Leaflet for Informed Consent

项目名称：居家艾灸改善腹膜透析相关性疲倦的疗效与安全性：一项随机对照预试验

申办者/课题下达单位：广东省中医院

伦理审查批件号：广东省中医院伦理委员会 BF2022-229-01

同意声明

我已经阅读了上述有关本研究的介绍，而且有机会就此项研究与医生讨论并提出问题。我提出的所有问题都得到了满意的答复。

我知道参加本研究可能产生的风险和受益。我知晓参加研究是自愿的，我确认已有充足时间对此进行考虑，而且明白：

- 我随时可以向医生咨询更多的信息。
- 我可以随时退出本研究，而且不会受到歧视或报复，医疗待遇与权益不会受到影响。

我同样清楚，如果我中途退出本研究，特别是由于药物的原因使我退出研究时，我若将病情变化告诉医生，完成相应的体格检查和理化检查，这将对我本人和整个研究十分有利。

如果因患病我需要采取任何其他的药物治疗，我会在事先征求医生的意见，或在事后如实告诉医生。

我同意药品监督管理部门、伦理委员会或申办者代表查阅我的研究资料。

我将获得一份经过签名并注明日期的知情同意书副本。

最后，我决定同意参加本项研究。

受试者 签名：____________ 日期：______年____月____日 联系电话：______________

监护人/授权委托人 签名：____________ 与受试者关系：____________

（注：受试者因无/限制行为能力等原因不能签署知情同意的，由其监护人或授权委托人签署）

联系电话：______________ 日期：______年____月____日

我确认已向受试者解释了本试验的详细情况，包括其权利以及可能的受益和风险，并给其一份签署过的知情同意书副本。

研究者 签名：________________ 日期：________年_____月_____日

研究者的工作电话：________________ 手机号：________________

广东省中医院伦理委员会办公室联系电话：020-81887233-35943
