# Supplementary material for: The Feasibility of Home-Based Moxibustion for Fatigue in Peritoneal Dialysis: A Randomized Controlled Pilot Trial
Source: Healthcare (Basel). 2026 Sep 20;14(18):3110. doi: 10.3390/healthcare14183110 (PMC13606285; doi:10.3390/healthcare14183110)
Supplement: Supplementary file 1 [file healthcare-14-03110-s001.zip › Supplement2 Supplemental Online Content_20260924 1 1.pdf]

Supplemental 2. Supplemental Online Content
Feasibility of Home-Based Moxibustion for Fatigue in Peritoneal Dialysis: A Randomized Controlled Pilot Trial

Supplemental Figure S1. Structure of the Moxibustion Device Used in Home-Based Intervention
Supplemental Figure S2. Demonstration of Moxibustion Application at the Shenque (CV8) Acupoint
Supplemental Figure S3. Changes in Patient-Reported Outcomes Over Time by Group.
Supplemental Table S1. Study Inclusion and Exclusion Criteria
Supplemental Table S2. Primary and Secondary Outcomes
Supplemental Table S3. Nutritional and Laboratory Outcomes at Week 12 by Study Group
Supplemental Table S4. Between-Group Comparisons of PFS–R Total and Domain Scores, and Fatigue Severity Categories at each Time point
Supplemental Table S5 Between-Group Comparisons of Patient-Reported Outcomes at each Time point
Supplemental Table S6. Within-Group Estimated Changes From Baseline to Week 12 in Secondary Outcomes
Supplemental Table S7. Between-Group Differences in Change From Baseline to Week 12 in Secondary Outcomes
Supplemental Table S8. Repeated-Measures ANOVA for Time, Group, and Interaction Effects on Primary and Patient-Reported Outcomes

Supplemental Figure S1. Structure of the Moxibustion Device Used in Home-Based Intervention

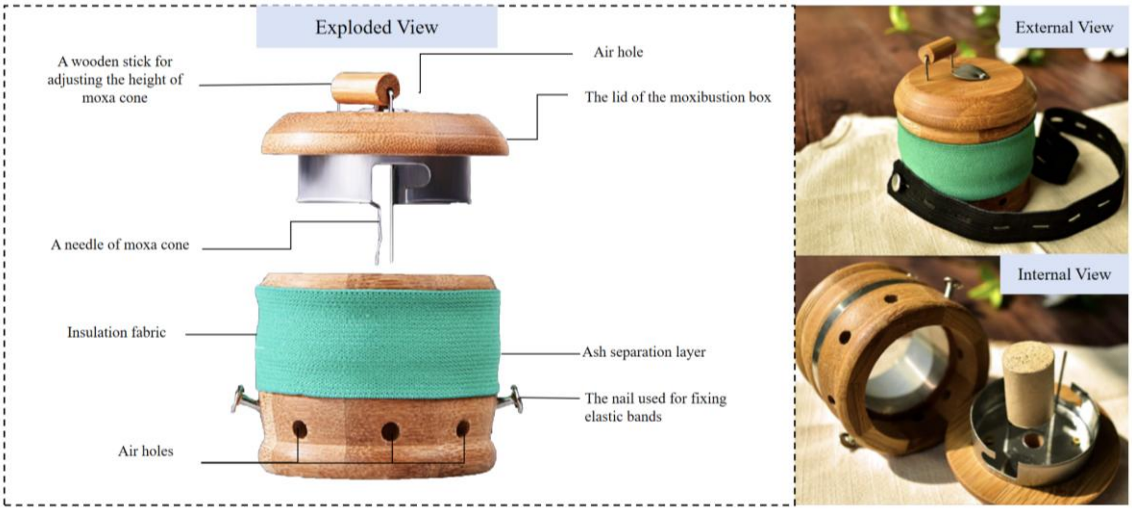

This figure illustrated the structural components of the standardized moxibustion device used in the home-based intervention. The exploded view displays the internal configuration of the device, including a wooden stick for adjusting the height of the moxa cone, a lid with an air hole for ventilation, a fixed needle for mounting the moxa cone, and an ash separation layer designed to prevent hot ash from falling directly

onto the skin. The lower portion of the device features a cylindrical bamboo shell with multiple air holes to ensure airflow and combustion efficiency, as well as external nails used for securing elastic bands to fasten the device to the abdomen. The insulation fabric encircling the main body provides thermal protection and reduces the risk of burns. The external and internal views depict the fully assembled device, demonstrating the relative positions of its components and how it appears in actual use.

Supplemental Figure S2. Demonstration of Moxibustion Application at the Shenque (CV8) Acupoint

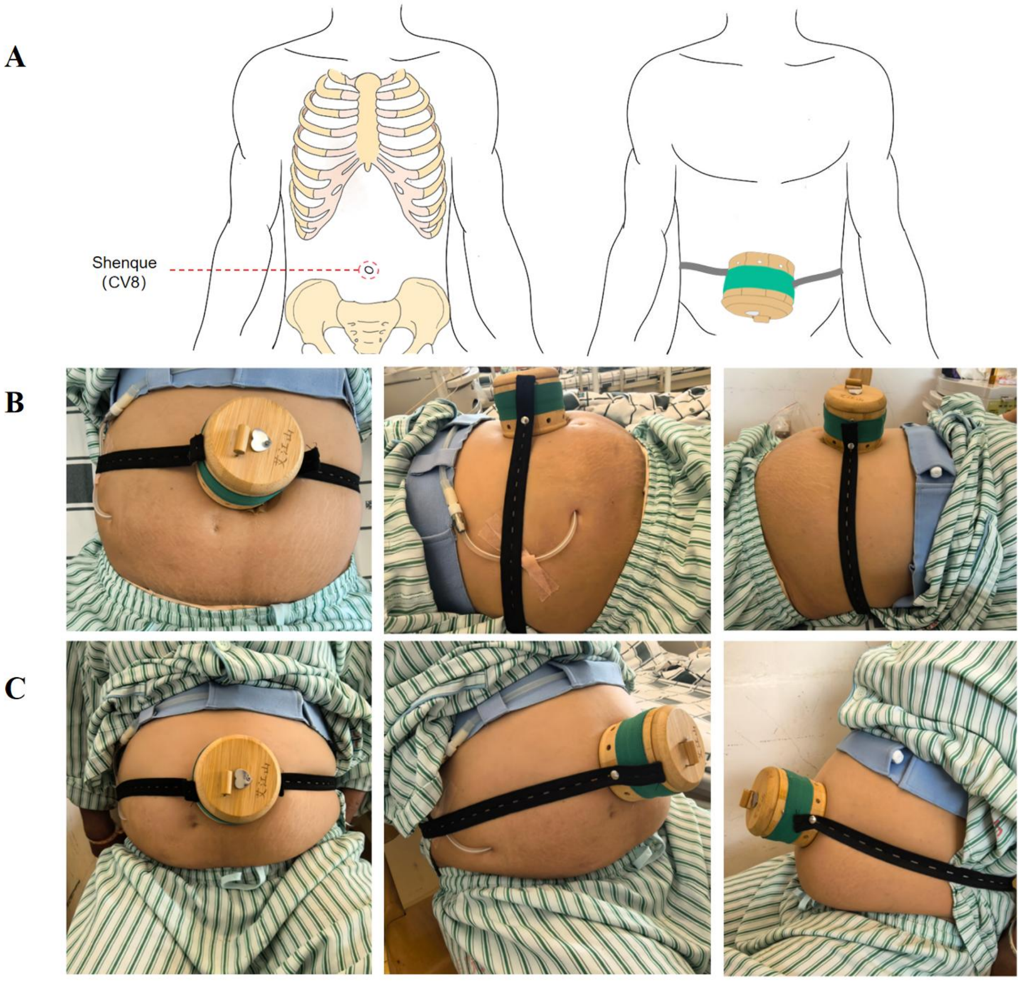

This figure illustrated the standardized application site and typical patient postures during home-based moxibustion therapy. (**A**) showed a schematic diagram of the Shenque (CV8) acupoint at the center of the umbilicus, which was the selected moxibustion site for all participants. (**B**) application of the moxibustion device in a supine position. (**C**) application of the moxibustion device in a seated position.

Supplemental Figure S3. Changes in Patient-Reported Outcomes Over Time by Group.

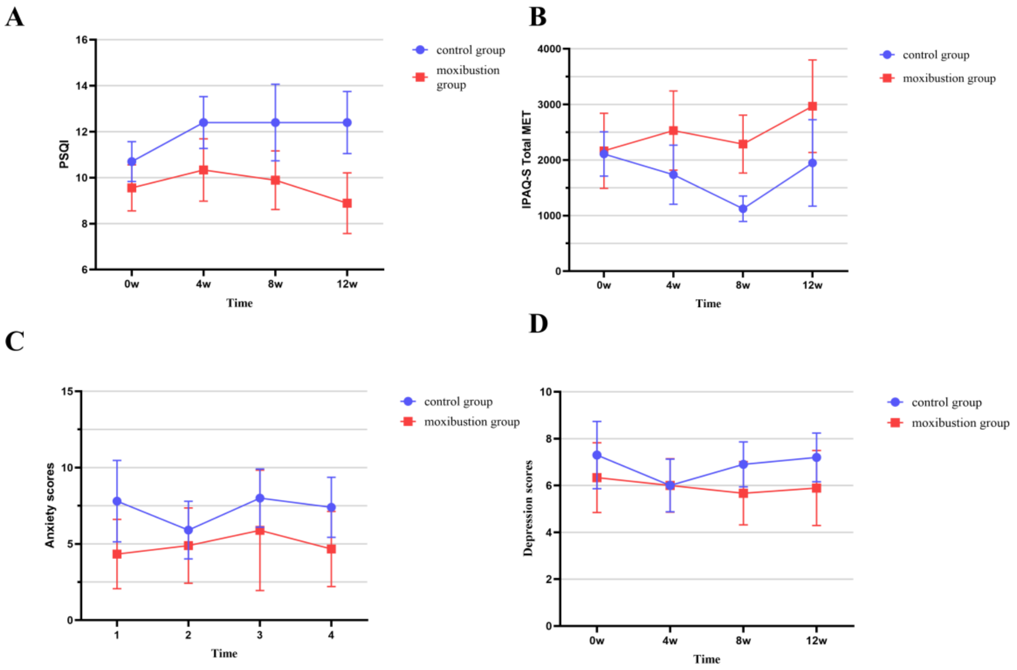

Figure legend: (A). Pittsburgh Sleep Quality Index (PSQI) Scores Over Time; (B). IPAQ-SF Total MET Scores Over Time; (C). HADS-Anxiety Scores Over Time; (D) HADS-Depression Scores Over Time.

Supplemental Table S1. Study Inclusion and Exclusion Criteria

**Inclusion criteria**	1. Adults aged 18 years or older;<br>2. Receiving maintenance peritoneal dialysis for at least 4 months and clinically stable condition prior to enrollment;<br>3. Reporting persistent fatigue symptoms for ⩾ 4 weeks and a total score of ⩾ 4 on the Revised Piper Fatigue Scale (PFS-R);<br>4. Capable of verbal communication, without cognitive or language impairment;<br>5. Willing to participate in the study and able to provide written informed consent.
**Exclusion criteria**	1. Life expectancy less than 6 months;<br>2. Receiving concurrent hemodialysis or with prior kidney transplantation;<br>3. Experiencing severe anemia, infection, an acute exacerbation of heart failure, or other comorbidities requiring recent adjustment of peritoneal dialysis regimen;<br>4. Having impaired nutritional intake or moderate-to-severe malnutrition (defined as serum albumin < 25 g/L);<br>5. Presence of local skin conditions, (e.g., lesions, rashes, ecchymosis,

	or petechiae) that may contraindicate moxibustion;<br>6. History of fatigue-targeted therapies or changes to the peritoneal dialysis prescription for fatigue relief within 4 weeks prior to enrollment;<br>7. Previous hypersensitivity or serious adverse reaction to moxibustion, or other reasons precluding adherence to the intervention protocol;<br>8. Co-existing medical conditions unrelated to end-stage renal disease that may contribute to fatigue (e.g., malignancy or other chronic wasting illnesses);<br>9. Concurrent participation in another clinical trial.

Supplemental Table S2. Primary and Secondary Outcomes

**Outcome category**	Definition
**Primary outcomes**	1. Between-group difference in mean overall Revised Piper Fatigue Scale (PFS-R) scoreat week 12.<br>2. Proportion of participants in the moxibustion group who achieved the target intervention dose (defined as completion of at least 3 home-based moxibustion sessions in each week for 12-week intervention period).
**Secondary outcomes**	1. Between-group differences at week 12 in laboratory indicators related to peritoneal dialysis adequacy and metabolic status, including serum creatinine, serum urea nitrogen, serum albumin, hemoglobin, calcium, phosphorus, parathyroid hormone, C-reative protein, weekly Kt/V, and total creatinine clearance .<br>2. Between-group differences in scores on the European Organization for Research and Treatment of Cancer Quality of Life Questionnaire Core 30 (EORTC QLQ-C30).<br>3. Between-group differences in sleep quality measured by the Pittsburgh Sleep Quality Index (PSQI).<br>4. Between-group differences in psychological distress measured by the Hospital Anxiety and Depression Scale (HADS).<br>5. Between-group differences in physical activity level assessed by the International Physical Activity Questionnaire–Short Form (IPAQ-SF).
**Additional feasibility outcomes**	1. Study retention;<br>2. Completion of scheduled on-site visits and telephone follow-ups;<br>3. Number of completed moxibustion sessions per participant; and dose intensity relative to the minimum prescribed dose.

**Safety outcomes**	1. Number and proportion of participants experiencing at least 1 adverse event;<br>2. Total number of adverse events;<br>3. Severity of adverse events;<br>4. Intervention relatedness of adverse events;<br>5. Adverse events resulting in temporary interruption or permanent discontinuation of the intervention.

Supplemental Table S3. Nutritional and Laboratory Outcomes at Week 12 by Study Group

	Control group (n=10)	Moxibustion group (n=9)	Difference (95% CI)	*P value*[ab]
**Nutritional Index**				
BMI, median (SD), kg/m²	20.77 (18.71, 22.82)	23.66 (21.93,25.40)	2.90 (0.38,5.42)	0.03
**Laboratory Parameters**				
Serum creatinine, median (IQR), mg/dL	11.55 (11.09, 12.85)	8.68 (7.84, 12.68)	-2.20 (-4.28, 1.38)	0.17
Serum urea nitrogen, median (IQR), mg/dL	64.50 (49.41,68.63)	58.24 (56.55,69.44)	1.90 (-11.32, 16.13)	0.74
Estimated GFR, median (IQR), mL/min/1.73m²	3.92 (3.32, 3.99)	5.10 (3.00, 5.34)	1.13 (-0.61, 1.75)	0.22
Hemoglobin, mean (SD), g/dL	11.49 (1.35)	11.60 (1.03)	0.11(-1.06,1.28)	0.85
Calcium, mean (SD), mg/dL	8.96 (0.55)	9.23 (0.72)	0.27(-0.35,0.89)	0.37
Phosphorus, median (IQR), mg/dL	5.11 (4.95, 5.33)	4.83 (4.80, 5.11)	-0.24 (-0.62, 0.15)	0.17
Parathyroid hormone, mean (SD), pg/mL	444.75 (312.89)	282.66 (195.11)	-162.09 (-418.10,93.91)	0.20
Serum albumin, mean (SD), g/dL	3.66 (0.44)	3.66 (0.36)	0.00 (-0.39,0.39)	>0.99
C-reactive protein, median (IQR), mg/dL	0.77 (0.01, 1.79)	3.52 (0.01, 5.43)	1.73 (-0.82, 5.42)	0.37

Abbreviations: eGFR, estimated glomerular filtration rate; SI conversion factor: To convert serum creatinine to umol/L, multiply by 88.4; Serm urea nitrogen to mmol/L, multiply by 0.357; Hemoglobin to g/L, multiply by 10. Calcium to mmol/L, multiply by 0.25; Phosphorus to mmol/L, multiply by 0.323; Parathyroid hormone to ng/L, multiply by 1; Serum albumin to g/L, multiply by 10.

(a) P values were calculated using the independent-sample t test for normally

distributed continuous variables, Mann–Whitney U test for nonnormally distributed continuous variables.
(b) All statistical tests were 2-sided with P < 0.05 was considered statistically significant.

Supplemental Table S4. Between-Group Comparisons of PFS–R Total and Domain Scores, and Fatigue Severity Categories at Each Time point

	Time point	Control group (n=10)	Moxibustion group (n=9)	*P-value*[a]
**PFS-R total score**[b]				
	0w	6.1 (1.2)	5.3 (1.0)	0.18
	4w	5.18 (1.30)	4.15 (1.20)	0.09
	8w	5.01 (1.15)	4.15 (1.04)	0.10
	12w	4.5 (1.3)	3.4 (0.8)	0.04
**PFS-R Domain score**[b]				
Behavioral	0w	5.7 (4.5, 7.2)	4.4 (3.7, 6.0)	0.11
	4w	5.4 (4.8, 6.2)	3.6 (3.0, 4.3)	0.11
	8w	5.2 (4.8, 6.7)	4.4 (3.6, 5.8)	0.10
	12w	4.8 (1.2)	3.9 (1.0)	0.12
Affective	0w	6.7 (4.0, 7.4)	6.6 (3.0, 7.0)	0.44
	4w	5.1 (4.4, 7.0)	4.2 (2.8, 5.0)	0.14
	8w	5.6 (4.0, 7.0)	2.8 (1.6, 3.6)	0.07
	12w	4.7 (2.4, 6.0)	2.4 (1.6, 4.2)	0.05
Sensory	0w	6.9 (6.8, 7.8)	7.0 (6.2, 7.8)	0.84
	4w	5.7 (4.0, 6.8)	6.4 (5.0, 6.6)	0.84
	8w	5.7 (3.4, 6.4)	4.6 (4.4, 6.0)	0.90
	12w	4.6 (3.6, 6.6)	3.8 (3.0, 4.6)	0.14
Cognitive	0w	5.9 (4.7, 7.0)	4.7 (4.3, 6.3)	0.44
	4w	5.0 (4.0, 6.3)	3.5 (3.0, 4.0)	0.03
	8w	5.0 (4.3, 6.2)	4.5 (3.5, 4.5)	0.20
	12w	4.4 (2.8, 4.8)	3.3 (2.8, 3.8)	0.16
**Fatigue Severity Categories**[c]				
Mild		0 (0)	0 (0)	
Moderate	0w	7 (70)	8 (89)	0.58
Severe		3 (30)	1 (11)	
Mild		2 (20)	2 (22)	
Moderate	4w	6 (60)	7(78)	0.63
Severe		2 (20)	0 (0)	
Mild		2 (20)	2 (22)	
Moderate	8w	8 (80)	7(78)	>0.99
Severe		0 (0)	0 (0)	
Mild		3 (30)	4 (44)	
Moderate	12w	7 (70)	5 (56)	0.65
Severe		0 (0)	0 (0)	

Abbreviations: PFS-R, Piper Fatigue Scale–Revised; SD, standard deviation; IQR,

interquartile range; w, week.
(c) P values were calculated using the independent-sample t test, Mann-Whitney U test or the Fisher exact test. All statistical tests were 2-sided with P < .05 was considered statistically significant.
(d) Values are present as mean (SD) for normally distributed variables and median (IQR) for non-normally distributed variables.
(e) Categorical variables are presented as number (percentage). Fatigue severity was categorized based on PFS-R total scores: mild (1–3), moderate (4–6), and severe (7–10).

Supplemental Table S5. Between-Group Comparisons of Patient-Reported Outcomes at Each Time point

Outcome	Time point	Control group (n=10)	Moxibustion group (n=9)	P-value[a]
**EORTC QLQ-C30[b]**				
	0w	41.6 (33.5, 44.8)	44.9 (39.9, 47.9)	0.19
	4w	39.4 (5.4)	40.8 (4.7)	0.58
	8w	38.3 (6.6)	40.9 (7.6)	0.44
	12w	37.5 (34.8,44.0)	40.9 (38.2,45.8)	0.13
**PSQI score[b]**				
	0w	10.0 (9.0, 11.0)	10.0 (9.0, 12.0)	0.68
	4w	12.0 (9.0,16.0)	11.0(6.0,14.0)	0.27
	8w	14.5 (8.0,17.0)	9.0 (7.0, 13.0)	0.19
	12w	12.40 (4.27)	8.89 (3.95)	0.08
**HADS score[b]**				
Anxiety	0w	7.0 (6.0, 12.0)	3.0 (3.0, 6.0)	0.03
	4w	6.5 (4.0,8.0)	4.0 (3.0,6.0)	0.27
	8w	7.0 (6.0,10.0)	4.0 (3.0,7.0)	0.12
	12w	8.0 (7.0,9.0)	5.0 (2.0,7.0)	0.04
Depression	0w	7.3 (4.6)	6.3 (4.5)	0.65
	4w	6.0 (3.6)	6.0 (3.4)	>0.99
	8w	6.9 (3.0)	5.7 (4.1)	0.46
	12w	7.5 (5.0, 10.0)	4.0 (3.0, 7.0)	0.27
**Physical Activity Level[c]**				
High		3 (30)	2 (22)	
Moderate	0w	6 (60)	5 (56)	>0.99
Low		1 (10)	2 (22)	
High		1 (10)	3 (33)	
Moderate	4w	6 (60)	4 (44)	0.59
Low		3 (30)	2 (22)	
High		0 (0)	4 (44)	
Moderate	8w	9 (90)	3 (33)	0.01
Low		1 (10)	2 (22)	
High		3 (30)	2 (22)	
Moderate	12w	4 (40)	6 (67)	0.59
Low		3 (30)	1 (11)	

Abbreviations: EORTC QLQ-C30, European Organization for Research and Treatment of Cancer Quality of Life Questionnaire-Core 30; PSQI, Pittsburgh Sleep Quality Index; HADS, Hospital Anxiety and Depression Scale; IPAQ-SF, International Physical Activity Questionnaire–Short Form; SD, standard deviation; IQR, interquartile range; w, week.

(a) P values were calculated using the independent-sample t test, Mann-Whitney U test or the Fisher exact test. All statistical tests were 2-sided with $P < 0.05$ was considered statistically significant.

(b) Values were expressed as mean (SD) for normally distributed variables and median (IQR) for non-normally distributed variables.

(c) Categorical variables are presented as number (percentage). Physical activity levels were categorized as low, moderate, or high based on total metabolic equivalent task (MET) minutes per week, calculated from responses to the IPAQ-SF, following standard scoring manual.

Supplemental Table S6. Within-Group Estimated Changes From Baseline to Week 12 in Ssecondary Outcomes

	Control group (n=10)		Moxibustion group (n=9)	
	**Change From Baseline[a] (95% CI)**	**P-value[b]**	**Change From Baseline[a] (95% CI)**	**P-value[b]**
**Nutritional Index**				
BMI, kg/m^2	0.34 (-0.13, 0.80)	0.14	0.20 (-0.88, 1.29)	0.68
**Dialysis Index**				
4-hour D/P	-0.05 (-0.13, 0.03)	0.19	-0.05 (-0.09, -0.01)	0.03
Total Kt/V	0.05 (-0.28, 0.37)	0.76	-0.01 (-0.49, 0.47)	0.96
Residual kidney Kt/V	-0.02 (-0.15, 0.11)	0.76	-0.12 (-0.42, 0.17)	0.37
Peritoneal Kt/V	0.06 (-0.21, 0.34)	0.60	0.11(-0.14, 0.36)	0.34
Total CCr, L/week	-2.10 (-8.15, 3.94)	0.45	4.15 (-13.80, 22.10)	0.61
Residual kidney CCr, L/week	-1.49 (-6.33, 3.36)	0.51	0.13 (-15.21, 15.47)	0.99
Peritoneal CCr, L/week	-0.62 (-4.97, 3.74)	0.76	4.02 (-3.35, 11.39)	0.24
**Laboratory Parameters**				
Serum creatinine, mg/dL	0.91 (0.44, 1.37)	**0.002**	0.48 (-0.70, 1.67)	0.37
Serum urea nitrogen, mg/dL	3.37 (-5.11, 11.85)	0.39	-3.13(-12.81, 7.27)	0.77
Estimated GFR, mL/min/1.73m^2	-0.51 (-0.78, -0.24)	**0.002**	-0.37 (-1.17, 0.44)	0.32
Hemoglobin, g/dL	-0.23 (-1.78, 1.32)	0.75	0.52 (-0.38, 1.43)	0.22
Calcium, mg/dL	-0.52 (-1.00, -0.05)	0.04	-0.40 (-0.74, 0.36)	0.17
Phosphorus, mg/dL	0.25 (-0.42, 0.92)	0.43	0.19 (-0.42, 0.80)	0.49
Parathyroidhormone, pg/mL	22.40(-176.13, 123.88)	0.72	-31.96(-224.39, 160.48)	0.71
Serum albumin,g/dL	-0.02 (-0.45, 0.42)	0.94	0.15 (-0.17, 0.46)	0.32
**Patient-Reported Outcome**				

	Control group (n=10)		Moxibustion group (n=9)	
	**Change From Baseline[a] (95% CI)**	**P-value[b]**	**Change From Baseline[a] (95% CI)**	**P-value[b]**
EORTC QLQ-C30 score	-4.68 (-8.25, 11.03)	0.51	-1.28 (-12.99, 4.36)	0.31
PSQI score	2.00 (-3.00, 7.00)	0.22	-2.00 (-5.00, 4.00)	0.63
HADS-Anxiety score	1.00 (-4.00, 4.00)	0.88	-1.00 (-1.00, 2.00)	0.67
HADS-Depression score	-0.1 (-4.00, 3.80)	0.95	0.00 (-3.00, 2.00)	0.72

Abbreviations: BMI, body mass index; Kt/V, weekly urea clearance normalized to the volume of distribution of urea; CCr, creatinine clearance; D/P, dialysate/plasma creatinine ratio; eGFR, estimated glomerular filtration rate; CI, confidence interval.

(a) Positive values indicate an increase from baseline; negative values indicate a decrease from baseline. Values represent within-group changes from baseline to week 12 and were presented as mean differences with 95% CIs.

(b) Statistical significance was calculated using paired t test or paired Wilcoxon test, depending on data distribution. P values < .05 were considered statistically significant.

Supplemental Table S7. Between-Group Differences in Change From Baseline to Week 12 in Secondary Outcomes

	Change From Baseline of Control Group (95% CI)(n=10)	Change From Baseline of Moxibustion Group(95% CI) (n=9)	Between-Group Difference in Change[a] (95% CI)	P-value[a]
**Nutritional Index**				
BMI, kg/m^2	0.34 (-0.13, 0.80)	0.20 (-0.88, 1.29)	-0.15 (-1.29, 0.90)	0.650.79
**Dialysis Index**				
4-hour D/P	-0.05 (-0.13, 0.03)	-0.05 (-0.09, -0.01)	0.00 (-0.08, 0.09)	0.97
Total Kt/V	0.05 (-0.28, 0.37)	-0.01 (-0.49, 0.47)	0.07 (-0.44, 0.47)	0.81
Residual kidney Kt/V	-0.02 (-0.15, 0.11)	-0.12 (-0.42, 0.17)	-0.10 (-0.39, 0.18)	0.46
Peritoneal Kt/V	0.06 (-0.21, 0.34)	0.11(-0.14, 0.36)	0.08 (-0.28, 0.39)	0.68
Total CCr, L/week	-2.10 (-8.15, 3.94)	4.15 (-13.80, 22.10)	6.26 (-10.38, 22.89)	0.44
Residual kidney CCr, L/week	-1.49 (-6.33, 3.36)	0.13 (-15.21, 15.47)	1.62 (-12.49, 15.72)	0.81
Peritoneal CCr, L/week	-0.62 (-4.97, 3.74)	4.02 (-3.35, 11.39)	4.64 (-3.05, 12.33)	0.22
**Laboratory Parameters**				
Serum creatinine, mg/dL	0.91 (0.44, 1.37)	0.48 (-0.70, 1.67)	-0.42 (-1.54, 0.70)	0.44
Serum urea nitrogen, mg/dL	3.37 (-5.11, 11.85)	-3.13(-12.81, 7.27)	-4.73(-15.22, 5.77)	0.36
Estimated GFR, mL/min/1.73m^2	-0.51(-0.78, -0.24)	-0.37(-1.17, 0.44)	0.14 (-0.60, 0.89)	0.69
Hemoglobin, g/dL	-0.23(-1.78, 1.32)	0.52(-0.38, 1.43)	0.75(-0.97, 2.47)	0.37
Calcium, mg/dL	-0.52(-1.00, -0.05)	-0.40(-0.74, 0.36)	0.36 (-0.32, 0.88)	0.29
Phosphorus, mg/dL	0.25(-0.42, 0.92)	0.19(-0.42, 0.80)	-0.05 (-0.90, 0.79)	0.89
Parathyroid hormone, pg/mL	22.40 (-176.13, 123.88)	-31.96 (-224.39, 160.48)	-23.15 (-198.10, 202.10)	0.68
Serum albumin, g/dL	-0.02(-0.45, 0.42)	0.15(-0.17, 0.46)	0.15 (-0.28, 0.67)	0.35

z

Supplemental Table S7. Between-Group Differences in Change From Baseline to Week 12 in Secondary Outcomes (continued)

	Change From Baseline of Control Group (95% CI)(n=10)	Change From Baseline of Moxibustion Group(95% CI) (n=9)	Between-Group Difference in Change[a] (95% CI)	P-value[b]
**Patient-Reported Outcome**				
EORTC QLQ-C30 score	-4.68 (-8.25, 11.03)	-1.28 (-12.99, 4.36)	-0.41 (-12.30, 6.88)	0.97
PSQI score	2.00 (-3.00, 7.00)	-2.00 (-5.00, 4.00)	-3.00 (-7.00, 2.00)	0.25
HADS-Anxiety score	1.00 (-4.00, 4.00)	-1.00 (-1.00, 2.00)	0.00 (-3.00, 3.00)	>0.99
HADS-Depression score	-0.1 (-4.00,3.80)	0.00 (-3.00, 2.00)	-1.00 (-4.00, 3.00)	0.53

Abbreviations: BMI, body mass index; Kt/V, weekly urea clearance normalized to the volume of distribution of urea; CCr, creatinine clearance; D/P, dialysate/plasma creatinine ratio; eGFR, estimated glomerular filtration rate; CI, confidence interval.

(a) Between-group differences were calculated as the moxibustion group minus the control group. Mean differences in individual change scores are reported for normally distributed variables; location-shift estimates are reported for nonnormally distributed variables. Because within-group and between-group nonparametric estimates were calculated separately, the between-group estimate may not equal the arithmetic difference between the 2 within-group point estimates.

Supplemental Table S8. Repeated-Measures ANOVA for Time, Group, and Interaction Effects on Primary and Patient-Reported Outcomes

Outcomes	Time effect		Group effect		Interaction effect	
	**F**	**P value**	**F**	**P value**	**F**	**P value**
**PFS-R**	11.74	<0.001	5.98	0.03	0.16	0.86
**EORTC QLQ-C30**	1.46	0.24	1.56	0.23	0.57	0.64
**PSQI**	0.191	0.67	2.54	0.13	1.54	0.23
**IPAQ-SF**	0.75	0.53	1.61	0.22	0.48	0.70
**HADS**						
Anxiety	1.11	0.36	4.24	0.06	0.74	0.54
Depression	0.34	0.80	0.35	0.56	0.14	0.72

Abbreviations: PFS-R, Revised Piper Fatigue Scale; QLQ-C30, EORTC Core Quality of Life Questionnaire; PSQI, Pittsburgh Sleep Quality Index; IPAQ-SF, International Physical Activity Questionnaire–Short Form; HADS, Hospital Anxiety and Depression Scale.

Note: Repeated-measures analysis of variance (ANOVA) was used to test for time effect (across baseline, 4, 8, and 12 weeks), group effect (moxibustion vs control), and time-by-group interaction. P values < .05 were considered statistically significant. No adjustment was made for multiple comparisons because these analyses were exploratory.
